# Supplementary material for: A population-based retrospective study comparing cancer mortality between Moluccan migrants and the general Dutch population: equal risk 65 years after immigration?
Source: BMJ Open. 2019 Aug 15;9(8):e029288. doi: 10.1136/bmjopen-2019-029288 (PMC6701674; doi:10.1136/bmjopen-2019-029288)
Supplement: Author's manuscript [file bmjopen-2019-029288.draft_revisions.pdf]

BMJ Open

BMJ Open is committed to open peer review. As part of this commitment we make the peer review history of every article we publish publicly available.

When an article is published we post the peer reviewers' comments and the authors' responses online. We also post the versions of the paper that were used during peer review. These are the versions that the peer review comments apply to.

The versions of the paper that follow are the versions that were submitted during the peer review process. They are not the versions of record or the final published versions. They should not be cited or distributed as the published version of this manuscript.

BMJ Open is an open access journal and the full, final, typeset and author-corrected version of record of the manuscript is available on our site with no access controls, subscription charges or pay-per-view fees (http://bmjopen.bmj.com).

If you have any questions on BMJ Open's open peer review process please email info.bmjopen@bmj.com

BMJ Open

A population-based retrospective study investigating cancer mortality among Moluccan migrants in the Netherlands: equal risk 65 years after immigration?

Journal:	*BMJ Open*
Manuscript ID	bmjopen-2019-029288
Article Type:	Research
Date Submitted by the Author:	20-Jan-2019

Complete List of Authors:	van der Wal, Junus; Amsterdam UMC, location AMC, Department of Public Health<br>Bodewes, Adee; Amsterdam UMC, location AMC, Department of Public Health<br>Agyemang, Charles; Amsterdam UMC, location AMC, Department of Public Health<br>Kunst, Anton; Amsterdam UMC, location AMC, Department of Public Health

Keywords:	cancer, mortality, ethnicity, Epidemiology < ONCOLOGY, the netherlands, moluccans

SCHOLARONE™
Manuscripts

A population-based retrospective study investigating cancer mortality among Moluccan migrants in the Netherlands: equal risk 65 years after immigration?

Junus M. van der Wal, Adee. J. Bodewes, Charles Agyemang, Anton E. Kunst

Authors affiliation: Amsterdam UMC, University of Amsterdam, Department of Public Health, Meibergdreef 9, 1105 AZ Amsterdam, Netherlands

Corresponding author: Junus M. van der Wal (j.m.vanderwal@amc.uva.nl / +31641889849)

Word count: 3011

Key words: cancer, mortality, ethnicity, the Netherlands, Moluccans, generation

Author contribution

JW: contributed to the design of the study, analyzed the data presented in this article and wrote the first draft of the article.

AB: contributed to the design of the study, lead the acquisition of the data, assisted in the analysis of the data and was involved in drafting the manuscript.

CA: contributed substantially to the drafting of the manuscript.

AK: contributed to the design of the study, assisted in the analysis of the data and commented on previous drafts of the manuscript.

For peer review only - http://bmjopen.bmj.com/site/about/guidelines.xhtml

Funding

This work was supported by the Dutch organization BUAT (platform for Moluccans) in order to obtain data from the Dutch national cause of death registry.

Competing interest

The authors declare that they have no conflict of interest.

Ethical approval:

According to the Medical Ethics Review Committee (METC) of the Amsterdam Medical Centre, an official approval of this study was not required because the act on Medical Research Involving Human Subjects (WMO) does not apply to this study (METC correspondence number W15-201#15.0240). The data was analyzed anonymously at Statistics Netherlands (CBS) computers, where all possible measures were taken to protect the privacy of subjects.

Availability of data and materials:

The data that support the findings of this study are available from CBS but restrictions apply to the availability of these data, which were used under license for the current study, and so are not publicly available.

Acknowledgments

We thank the Dutch organization BUAT (Platform for Moluccans) for contributing financially to the acquisition of data from the Dutch national cause of death registry. Furthermore, we thank CBS for their aid in obtaining the mortality and demographic data.

For peer review only

Abstract

Objective – To the hypothesis that cancer mortality rates among the Moluccan-Dutch, the oldest non-Western migrant group in the Netherlands, are similar to those in the general Dutch population.

Design – Population-based retrospective study.

Setting – Data from the national cause of death registry in the Netherlands and municipal registries.

Participants – Using historic records containing family names of all Moluccan-Dutch who arrived in the Netherlands in 1951, we identified 35,581 Moluccan-Dutch persons in the national cause of death registry of the Netherlands. The reference group consisted of 9,064,169 persons of the general Dutch population.

Outcome measures – Mortality data were linked to demographic data from municipal registries. We calculated all-cancer and cancer-specific mortality and measured differences between the two groups using Poisson regression, adjusting for sex, age and area socioeconomic status. We conducted a sub-analysis for the first and second generation Moluccan-Dutch.

Results – There was no difference in all-cancer mortality between Moluccan-Dutch and the general Dutch population. Mortality was higher among Moluccan-Dutch for liver, cervix and corpus uteri cancers, but lower for stomach, esophagus, kidney and nervous system cancers. For most cancers, mortality risk as compared to the general Dutch population varied between different generations of Moluccan-Dutch.

Conclusions – Several decades after migration, the Moluccan-Dutch show similar all-cancer mortality, but different cancer-specific mortality rates, when compared to the general Dutch population.

For peer review only - http://bmjopen.bmj.com/site/about/guidelines.xhtml

Article summary

Strengths and limitations of this study

- This is the first study to investigate cancer mortality among the Moluccan-Dutch, the oldest non-western migrant group to enter the Netherlands after the second world war.
- We employed a novel approach to select persons from Moluccan descent from the Dutch national cause-of-death registry, by selecting persons based on the Moluccan family names registered in historical immigration documents, which were made available to us for research purposes.
- A limitation of this novel selection method is the fact the we were unable to identify second generation Moluccan-Dutch persons with a native Dutch father and consequently Dutch family name.
- Another limitation is the low number of deaths for most cancer types in the Moluccan-Dutch group, limiting statistical power.

For peer review only

For peer review only - http://bmjopen.bmj.com/site/about/guidelines.xhtml

Introduction

Ethnic disparities in the prevalence and mortality burden of different cancer types are known to exist in Europe.[1] For instance, migrants from low- and middle-income countries (LMIC) tend to show lower all-cancer mortality and morbidity. Moreover, cancer burden among LMIC-migrants is thought to be influenced by differences in cancer epidemiology between high-income countries (HICs) and LMIC and exposure to concordant risk factors.[2] Indeed, when compare to their host population, cancers related to infection, such as liver, stomach and cervical cancer, are more common in migrants from LMICs; cancers considered to be related to Western lifestyle such as colorectal, breast and esophageal cancer are less common in these groups.[1]

Increasing evidence suggests that such epidemiological differences become less pronounced as LMIC-migrants reside in their destination country for a longer period, as local (Western) cancer-related risk factors will eventually predominate over risk factors associated with their country of origin. Moreover, second generation migrants who were born in HICs lack early life risk factor exposures experienced by the first generation, such as possible higher infection pressure.[3] Accordingly, some studies on LMIC-migrants who have resided in their host country for more than one generation have suggested a shift in cancer epidemiology towards rates of the native population of their destination country.[4–6] However, given the limited number of studies on this topic, more research is necessary to further characterize the cancer risk profile in these groups and address the question to what extent ethnic disparities in cancer mortality persist or start to converge to those of the host population.

For this reason, we set out to assess the difference in cancer mortality between the general Dutch population and the Moluccan-Dutch, the oldest LMIC migrant group to settle in the Netherlands after the Second World War. The Moluccan-Dutch are a migrant group from the Moluccan Islands in Indonesia that settled in the Netherlands in 1951. Earlier studies found differences in cancer standard incidence ratios and mortality between migrants from Indonesia and the general Dutch population.[7–9]

For peer review only - http://bmjopen.bmj.com/site/about/guidelines.xhtml

In our view, separate analysis of the Moluccan-Dutch is desirable, since they are a homogenous group within the Indonesian community with their own cultural practices, lifestyle and migration history, all of which can have a profound impact on cancer risk.

The aim of this study was to assess the difference in all cancer and cancer specific mortality rates between the Moluccan-Dutch and the general Dutch population, with a sub-analysis for the first and second generation. Since the Moluccan-Dutch have resided in the Netherlands for over 65 years, we expect local environmental factors in the Netherlands to reflect on mortality rates in cancers that are known to be influenced by factors such as lifestyle and exposure to infectious agents. Therefore, we hypothesize cancer mortality rates to be similar to those of the host population, especially for the second generation.[4,6]

For peer review only - http://bmjopen.bmj.com/site/about/guidelines.xhtml

For peer review only

Methods

Study population

The Moluccan-Dutch are a migrant group consisting of the former soldiers from the Royal Netherlands East Indies Army (KNIL), the military force of the Netherlands in their former colony, and their families, who migrated to the Netherlands in spring 1951 after decolonization of Indonesia. Even though their stay was supposed to be temporary, the majority settled in the Netherlands. No considerable chain- or remigration occurred, resulting in a fairly static minority group. The integration of the first generation was difficult, mainly because their stay was thought to be temporary and because of societal tensions resulting from their ongoing struggle to found their own republic on the Moluccan Islands. The second generation prospered when compared to the first, but there are signs that integration of the third generation faltered, especially when considering degree of education. However, the Moluccan-Dutch are presently found to be fairly well integrated in Dutch society.[10]

Data sources and handling

For this study we linked data from the cause of death registry of Statistics Netherlands (CBS) to demographic data from municipal registries, including date of birth, sex and place of residence. Data were extracted based a person's surname. Upon arrival in the Netherlands in 1951, all Moluccan families were registered by the government in documents that were made available to us in order to identify persons of Moluccan descent from the cause of death registry. Since Moluccans carry very distinctive names within Indonesia, overlap with other Indonesian (or non-Indonesian) persons was deemed unlikely. After identification in registries, data were anonymized. Area-level social economic status (SES) was determined based on decile scores calculated by the Netherlands Institute for Social Research (SCP), reflecting SES of inhabitants of specific postal codes. A higher decile score indicates a higher area SES.

For peer review only - http://bmjopen.bmj.com/site/about/guidelines.xhtml

We used birth year to stratify our study population in first and second generation, since we had no information on country of birth. We considered people born up to 1951, the year of migration, to be of the first generation and people born after 1951 but before 1970 of the second generation. We chose 1970 as cutoff point since Moluccan-Dutch born after this year will increasingly be offspring of the second generation (i.e. of the third generation). Furthermore, we created two groups within the first generation, based on whether the person was an adult (born before 1934) or a minor (born between 1934 and 1951) in the year that the Moluccan migrants were transported to the Netherlands. The reference group, referred to as 'general Dutch population', consisted of 9 064 169 persons with Dutch nationality.

Data analysis

All-cancer mortality encompassed codes C00-D48 in the 10th revision of the International Statistical Classification of Diseases and Related Health Problems (ICD-10). Furthermore, we chose to investigate the most common types of cancer in both HICs and LMICs, as identified by the global cancer research project GLOBOCAN 2012.[11] An overview of the types of cancers included in this study and their corresponding ICD-10 codes is listed in table 2. In breast cancer, we only included female patients due to low number of deaths among males for this cancer type.

We present both absolute numbers of deaths, as well as age-adjusted death rates, using the direct method of standardization. In accordance with CBS guidelines, number of deaths lower than ten are not shown. Poisson regression analysis was used to investigate the size of difference in mortality attributable to the different types of cancer between Moluccan-Dutch and the general Dutch population. Furthermore, we performed Poisson regression analysis for each generation and sex. Based on this regression analysis, we present relative risks (RR) with 95% confidence intervals (CI). Analyses were performed using IBM SPSS Statistics 20.

For peer review only - http://bmjopen.bmj.com/site/about/guidelines.xhtml

Results

Demographic characteristics of our study population are presented in table 1. A total of 81 591 Moluccan-Dutch persons were included in this study. Within the Moluccan-Dutch group, 15.7% was of the first generation (5.7% migrated as adult and 10.0% as minor), 27.9% was of the second generation and 56.4% was of the second or third generation. The Moluccan-Dutch in our study population showed higher rates in the lower SES groups, when compared to the general Dutch population.

An overview of total number of deaths and age-adjusted death rates for all different cancer types, as well as all-cancer mortality, is shown in table 2. In both the Moluccan-Dutch and the general Dutch population group, highest age-adjusted death rates were found for lung cancer, followed by colorectal and breast cancer, respectively.

Relative risk (RR) of cancer mortality for each investigated cancer type is presented in table 3. The Moluccan-Dutch showed similar all-cancer mortality risk when compared to the general Dutch population (RR = 0.98, CI: 0.93 – 1.03). However, Moluccan-Dutch men showed lower all-cancer mortality than men in the general Dutch population (RR = 0.92, CI: 0.85 – 0.99). Furthermore, the Moluccan-Dutch showed lower relative risk for mortality caused by cancer of the stomach (RR = 0.71, CI: 0.50-1.00), esophagus (RR = 0.46, CI: 0.31 – 0.68), kidneys (RR = 0.61, CI: 0.39 – 0.96) and nervous system (RR = 0.32, CI: 0.18 – 0.58), when compared to the general Dutch population. In contrast, they showed higher relative risk for mortality caused by cancer of the liver (RR = 2.60 CI: 1.98 – 3.42), cervix (RR = 1.89 CI: 1.13 – 3.14) and corpus uteri (RR = 1.72, CI: 1.12 – 2.64). Additionally, only Moluccan-Dutch men showed lower mortality risk for cancer of the stomach (RR = 0.57, CI: 0.35 – 0.91) and pancreas (RR = 0.63, CI: 0.42 – 0.95) and higher mortality risk for non-Hodgkin lymphoma (NHL) (RR = 1.60, CI: 1.13 – 2.27), when compared to men in the general Dutch population.

For peer review only - http://bmjopen.bmj.com/site/about/guidelines.xhtml

A regression analysis per generation within the Moluccan-Dutch group is presented in table 4. Among the first-generation adults, stomach cancer mortality was lower (RR = 0.48. CI: 0.26 – 0.89), but NHL mortality was higher (RR = 1.68, CI: 1.13 – 2.51), when compared to the general Dutch population. Furthermore, esophageal cancer mortality was lower in the first generation adults (RR = 0.37, CI: 0.17 – 0.82) and minors (RR = 0.39, CI: 0.21 – 0.76), whereas liver cancer mortality was higher among the first generation adults (RR = 3.96, CI: 2.74 – 5.70) and minors (RR = 1.79, CI: 1.06 – 3.03), when compared to their counterparts in the general Dutch population. Significantly elevated among the second generation, but not among first generation, were cancer of the cervix (RR = 2.39, CI: 1.28 – 4.47), breast (RR = 1.29, CI: 1.01 – 1.65), corpus uteri (RR = 3.14, CI: 1.56 – 6.33) and ovaries (RR = 1.76, CI: 1.09 – 2.83) and leukemia (RR = 1.95, CI: 1.17 – 3.25). Cancer of the brain and nervous system carried lower mortality among both first-generation minors (RR = 0.39, CI: 0.16 – 0.93) and the second generation Moluccans (RR = 0.25, CI: 0.09 – 0.66), when compared to the general Dutch population.

Two cancer types show inverse outcomes when comparing mortality risk of two consecutive generations of Moluccan-Dutch with the general Dutch population. Prostate cancer mortality was higher among first generation adults in the Moluccan-Dutch group when compared to their counterparts in the general Dutch population (RR = 1.61, CI: 1.28 – 2.03), but lower among first generation minors (RR = 0.45, CI: 0.22 – 0.90). Additionally, lower risk of dying of lung cancer was observed among first generation minors when compared to the general Dutch population (RR = 0.70, CI: 0.58 – 0.85), but the second-generation Moluccan-Dutch showed a higher risk (RR = 1.42, CI: 1.20 – 1.69).

For peer review only

For peer review only - http://bmjopen.bmj.com/site/about/guidelines.xhtml

Discussion

Key findings

Our objective was to compare cancer mortality between the Moluccan-Dutch and the general Dutch population. We found similar all-cancer mortality, but several differences in cancer-specific mortality across both the first and second generation of Moluccan-Dutch.

Strengths and limitations

This is the first study that was able to investigate cancer mortality specifically among the Moluccan-Dutch, since we were provided with historic records on the migration of the Moluccan migrants to the Netherlands, enabling us to select participant based on family name. A limitation of this selection method is that we were unable to include second generation Moluccan-Dutch persons with a Dutch father and consequently Dutch family name, however it is unclear how this would affect our data. Another limitation of this study is the fact that , for most cancer types, the total number of deaths was small among the Moluccan-Dutch, limiting our ability to detect significant differences between Moluccan-Dutch and the general Dutch population.

Discussion of the key findings

In the Netherlands, there is one earlier study reporting on cancer mortality among first and second generation Indonesian immigrants, which includes persons from the Moluccan Islands.[12] There seem to be multiple similarities in cancer mortality disparities between Moluccan-Dutch and the Indonesian immigrant group as a whole in the Netherlands, when compared to their Dutch counterparts. Both groups showed lower stomach cancer mortality, similar breast cancer mortality and higher liver and uterine cancer mortality when compared to their Dutch counterparts. However, the Indonesian immigrant group as a whole showed lower lung and colorectal cancer mortality when compared to

For peer review only - http://bmjopen.bmj.com/site/about/guidelines.xhtml

native Dutch, whereas we found no differences between Moluccan-Dutch and the general Dutch population for these cancer types.

In Europe, a marked feature of cancer epidemiology in LMIC migrant groups is their lower all-cancer mortality risk.1 However, we found that the Moluccan-Dutch experience similar all-cancer mortality when compared to the general Dutch population. Even though men showed significantly lower all-cancer mortality when compared to men in the general Dutch population, the difference was small as compared to other LMIC migrants in the Netherlands.[4,6]

Another characteristic of cancer epidemiology in LMIC migrants in Europe is a higher prevalence of cancer related to infection (pathogen-driven) and a lower prevalence of cancers thought to be related to Western lifestyle (non-pathogen driven).[1] Our results imply that the Moluccan-Dutch do not fully cohere to this pattern. We will discuss all cancers for which we found a significant difference between Moluccan-Dutch and the general Dutch population, separately discussing pathogen-driven and non-pathogen-driven cancers, and the cancer types that carried the highest mortality rates among both groups.

In pathogen-driven cancers, we found higher mortality risk among Moluccan-Dutch for liver and cervical cancer and a lower mortality risk for stomach cancer, when compared to the general Dutch population.

- The higher liver cancer mortality among the Moluccan-Dutch, especially in the first

generation, might be reflective of early life exposure in Indonesia to higher burden of hepatitis B and C infection, the most important risk factor for liver cancer.[13] The fact that first generation migrants from Indonesia in the Netherlands show higher infectious hepatitis mortality than the general Dutch population further supports to this theory.[12] Another important risk factor for liver cancer is alcohol consumption. However, since a health survey among Moluccan-Dutch found their level of alcohol

For peer review only - http://bmjopen.bmj.com/site/about/guidelines.xhtml

consumption to be lower when compared to native Dutch, this risk factor is unlikely to explain the difference found.[14]

- Cervical cancer mortality was only significantly elevated among Moluccan-Dutch women of the second generation, when compared to their counterparts in the general Dutch population. This is in contrast with earlier research on LMIC-migrants in Europe where women of the first generation were at increased risk.[1,5] There is no data on the prevalence among Moluccan-Dutch women of the most important etiological factors for cervical cancer, specific strains of the sexually transmitted human papillomavirus.[15]
- The lower stomach cancer mortality among Moluccan-Dutch was mostly attributable to a significantly lower risk in first generation adults when compared to the general Dutch population. Lower prevalence in Indonesia of the main risk factor, colonization with the bacteria Helicobacter pylori (HP), might have had a protective effect in this group.[16,17]

In non-pathogen-driven cancers, we found that the Moluccan-Dutch carry higher mortality risk for cancer of the corpus uteri, but lower mortality risk for esophageal and kidney cancer and cancers of the nervous system.

- For cancer of the corpus uteri, the higher mortality risk among Moluccan-Dutch women when compared to women from the general Dutch population is probably not explained by an important risk factor, obesity, since obesity rate was found to be similar between the two groups.[14,18] Another important risk factor is (cumulative) estrogen exposure, but data on this topic in Moluccan-Dutch women is lacking.[19]
- For esophageal cancer, important risk factors are mostly related to Western lifestyle, such as smoking, alcohol consumption and obesity.[20] The Moluccan-Dutch show no significant difference in smoking or body mass index when compared to native Dutch, but they are thought to consume less alcohol, which could contribute to the results.[14]
- Kidney cancer mortality risk was lower among Moluccan-Dutch, when compared to the general

For peer review only - http://bmjopen.bmj.com/site/about/guidelines.xhtml

Dutch population. A known risk factor for kidney cancer is hypertension, which actually may be more prevalent among Moluccan-Dutch.[14,21] Other risk factors are mostly related to a Western lifestyle, such as smoking and obesity, but prevalence of these particular risk factors is thought to be similar between Moluccan-Dutch and native Dutch.[14,22]

- Cancer of the nervous system mortality risk was lower among Moluccan-Dutch than among the general Dutch population. To date, only a few factors are known to influence the incidence of gliomas, such as ionizing radiation exposure, history of atopic constitution and some genetic risk factors.[23,24] Whether these factors are able to explain the lower risk among the Moluccan-Dutch is unclear.

For peer review only

We found no difference in risk of mortality between the entire group of Moluccan-Dutch and the general Dutch population for the cancer types that carried the highest mortality rates.

- In lung cancer, Moluccan-Dutch of the second generation showed a higher risk of dying from lung cancer, when compared to the general Dutch population. Smoking is the most important risk factor of lung cancer.[25] As stated earlier, a health survey among Moluccan-Dutch found no difference in smoking between Moluccan-Dutch and native Dutch, but no stratification by generation was made.[14]
- In breast cancer, Moluccan-Dutch women of the second generation experienced increased mortality risk, when compared to the general Dutch population. Since migrant women are thought to show lower attendance at the national breast cancer screening program, lower detection rate in the early stages of breast cancer, lowering the chance of curable treatment options and consequently increase mortality, might contribute to the results.[26,27] However, this raises the question why first generation Moluccan-Dutch women do not show higher mortality risk for breast cancer.
- For colorectal cancer, there was no difference in the mortality risk between Moluccan-Dutch and the general Dutch population, which is in contrast with most other LMIC migrant groups in Europe.[1] One could speculate that the similar mortality risk detected in our study might be reflective of adaptation to Western lifestyle among the Moluccan-Dutch since 1951.[28]

For peer review only - http://bmjopen.bmj.com/site/about/guidelines.xhtml

In conclusion, several decades after migration from Indonesia to the Netherlands, the Moluccan-Dutch showed similar all-cancer mortality when compared to the general Dutch population, but differences exist in cancer-specific mortality between the two groups, in both the first and second generation. In liver and stomach cancer, an increased period of exposure to local risk factors in the Netherlands might have led to a shift in mortality towards rates of the general Dutch population. However, for most other cancer types, such a tendency was not detected, unlike some other studies in the Netherlands that included second generation migrants.[4,6] Furthermore, these results highlight the need for research aimed at characterizing the cancer profile of LMIC migrants in high-income countries in order to aid tailored preventative and diagnostic efforts.

For peer review only

For peer review only - http://bmjopen.bmj.com/site/about/guidelines.xhtml

References

1. Arnold, M., Razum, O. & Coebergh, J. W. Cancer risk diversity in non-western migrants to Europe: An overview of the literature. *Eur. J. Cancer* **46**, 2647–2659 (2010).

2. Gushulak, B. D. & MacPherson, D. W. The basic principles of migration health: Population mobility and gaps in disease prevalence. *Emerg. Themes Epidemiol.* **3**, 1–11 (2006).

3. Spallek, J., Zeeb, H. & Razum, O. What do we have to know from migrant ' s past exposures to understand their health status ? A life course approach. *Emerg. Themes Epidemiol.* **8**, 1–8 (2011).

4. Stirbu, I. *et al.* Cancer mortality rates among first and second generation migrants in the Netherlands: Convergence toward the rates of the native Dutch population. *Int. J. Cancer* **119**, 2665–2672 (2006).

5. Beiki, O., Allebeck, P., Nordqvist, T. & Moradi, T. Cervical, endometrial and ovarian cancers among immigrants in Sweden: Importance of age at migration and duration of residence. *Eur. J. Cancer* **45**, 107–118 (2009).

6. Williams, G. *et al.* Cancer incidence and mortality of Surinamese migrants in the Netherlands: In-between Surinamese and Dutch levels? *Cancer Causes Control* **24**, 1375–1383 (2013).

7. Arnold, M., Aarts, M. J., Van Der Aa, M., Visser, O. & Coebergh, J. W. Investigating cervical, oesophageal and colon cancer risk and survival among migrants in the Netherlands. *Eur. J. Public Health* **23**, 867–873 (2013).

8. Arnold, M. *et al.* Diverging breast and stomach cancer incidence and survival in migrants in the Netherlands, 1996-2009. *Acta Oncol. (Madr).* **52**, 1195–1201 (2013).

9. Visser, O. & van Leeuwen, F. E. Cancer risk in first generation migrants in North-Holland/Flevoland, The Netherlands, 1995-2004. *Eur. J. Cancer* **43**, 901–908 (2007).

10. van Amersfoort, H. The waxing and waning of a diaspora: Moluccans in the Netherlands, 1950-2002. *J. Ethn. Migr. Stud.* **30**, 151–174 (2004).

For peer review only

For peer review only - http://bmjopen.bmj.com/site/about/guidelines.xhtml

11. Torre, L. A. *et al.* Global Cancer Statistics, 2012. *CA a cancer J. Clin.* **65**, 87–108 (2015).

12. Ho, L., Bos, V. & Kunst, A. E. Differences in cause-of-death patterns between the native Dutch and persons of Indonesian descent in the Netherlands. *Am. J. Public Health* **97**, 1616–1618 (2007).

13. El-Serag, H. B. Epidemiology of viral hepatitis and hepatocellular carcinoma. *Gastroenterology* **142**, 1264–1273.e1 (2012).

14. Van Der Wal, J. M., Bodewes, A. J., Agyemang, C. O. & Kunst, A. E. Higher self-reported prevalence of hypertension among Moluccan-Dutch than among the general population of the Netherlands: Results from a cross-sectional survey. *BMC Public Health* **14**, (2014).

15. Schiffman, M., Castle, P., Jeronimo, J., Rodriguez, A. & Wacholder, S. Human papillomavirus and cervical cancer. *Lancet* **370**, 890–907 (2007).

16. Huang, H. *et al.* Estimation of Cancer Burden Attributable to Infection in Asia. *J. Epidemiol.* **25**, 626–638 (2015).

17. Blankenstein, M. Van *et al.* The prevalence of helicobacter pylori infection in the Netherlands. **48**, (2013).

18. Reeves, G. K. *et al.* Cancer incidence and mortality in relation to body mass index in the Million Women Study: Cohort study. *Br. Med. J.* **335**, 1134–1139 (2007).

19. Collaborative Group on Epidemiological Studies on Endmetrial Cancer. Endometrial cancer and oral contraceptives: An individual participant meta-analysis of 27 276 women with endometrial cancer from 36 epidemiological studies. *Lancet Oncol.* **16**, 1061–1070 (2015).

20. Thrift, A. P. The epidemic of oesophageal carcinoma: Where are we now? *Cancer Epidemiol.* **41**, 88–95 (2016).

21. De Back, T. R., Bodewes, A. J., Brewster, L. M. & Kunst, A. E. Cardiovascular health and related health care use of Moluccan-Dutch immigrants. *PLoS One* **10**, 1–11 (2015).

22. Weikert, S. & Ljungberg, B. Contemporary epidemiology of renal cell carcinoma: Perspectives of primary prevention. *World J. Urol.* **28**, 247–252 (2010).

For peer review only

For peer review only - http://bmjopen.bmj.com/site/about/guidelines.xhtml

23. Ostrom, Q. T. *et al.* The epidemiology of glioma in adults: A state of the science review. *Neuro. Oncol.* **16**, 896–913 (2014).

24. Maile, E. J., Barnes, I., Finlayson, A. E., Sayeed, S. & Ali, R. Nervous system and intracranial tumour incidence by ethnicity in England, 2001-2007: A descriptive epidemiological study. *PLoS One* **11**, 2001–2007 (2016).

25. Alberg, A. J., Brock, M. V., Ford, J. G., Samet, J. M. & Spivack, S. D. Epidemiology of lung cancer: Diagnosis and management of lung cancer, 3rd ed: American college of chest physicians evidence-based clinical practice guidelines. *Chest* **143**, 1–29 (2013).

26. Vermeer, B. & Van den Muijsenbergh, M. E. T. C. The attendance of migrant women at the national breast cancer screening in the Netherlands 1997-2008. *Eur. J. Cancer Prev.* **19**, 195–8 (2010).

27. Islanders, P. *et al.* Differences in Breast Cancer Stage, Treatment, and Survival by Race and Ethnicity. **163**, 1–7 (2003).

28. Rasool, S., Kadla, S. A., Rasool, V. & Ganai, B. A. A comparative overview of general risk factors associated with the incidence of colorectal cancer. *Tumor Biol.* **34**, 2469–2476 (2013).

For peer review only

For peer review only - http://bmjopen.bmj.com/site/about/guidelines.xhtml

List of abbreviations

LMIC: low- and middle income countries

HIC: high-income countries

KNIL: Royal Netherlands East Indies Army

CBS: Statistics Netherlands

SES: social economic status

SCP: Netherlands Institute for Social Research

ICD-10: 10th revision of the International Statistical Classification of Diseases and Related Health Problems

CI: confidence interval

RR: relative risk

NHL: non-Hodgkin lymphoma

For peer review only

For peer review only - http://bmjopen.bmj.com/site/about/guidelines.xhtml

Table 1: Demographic characteristics the Moluccan-Dutch and the Dutch general population				
	Moluccan-Dutch		Dutch general population	
	N	%	N	%
Sex				
Male	40062	49.1	7917402	49.9
Female	41529	50.9	7949136	50.1
Generation (birth year)				
1 adult (<1934)	4642	5.7	2480434	15,6
1 minor (1935-1951)	8185	10.0	2639408	16.6
2 (1951-1969)	22754	27.9	3944327	24,9
≥2 (1970 >)	46010	56.4	6802369	42,9
Decile score				
1 (lowest SES[a])	7017	8.6	729861	4.6
2	7669	9,4	951992	6.0
3	10363	12.7	1729453	10.9
4	11341	13.9	1745319	11.0
5	11096	13.6	2491047	15.7
6	6935	8.5	1919851	12.1
7	6283	7.7	1856385	11.7
8	4732	5.8	1174124	7.4
9	7262	8.9	1586654	10
10 (highest SES)	8648	10.6	1650120	10.4
Missing	245	0.3	31734?	0.2
Total	81591	100	15866538	100

a: SES = socioeconomic status

For peer review only

For peer review only - http://bmjopen.bmj.com/site/about/guidelines.xhtml

Table 2: Absolute numbers of death and age-standardized death rates per cancer type

		Absolute numbers of death		Age-standardized death rates per 100,000	
Type of cancer	ICD-10[a] code	Dutch general population	Moluccan-Dutch	Dutch general population	Moluccan-Dutch
Stomach	C16	18443	33	4.98	3.51
Liver cell	C22	7227	53	2.03	6.37
Cervix uteri	C53	2488	15	0.77	1.51
Colorectal	C18-21	59548	138	16.11	15.51
Breast	C50	41324	134	12.59	13.20
Prostate	C61	30975	79	7.60	10.93
Bladder	C67	14875	27	3.86	2.91
Lung and lower airway	C33-34	119520	336	34.67	34.89
Esophagus	C15	18683	25	5.51	2.55
Corpus uteri	C54	5189	22	1.38	2.15
Ovaries	C56	12239	40	3.52	3.76
Pancreas	C25	27351	62	7.64	6.95
Kidney	C64	11491	20	3.24	1.98
Leukemia	C91-95	14349	48	3.71	5.04
Non-Hodgkin lymphoma	C82-85, C96	13620	48	3.70	5.13
Nervous-system	C70-72	10893	19	3.36	1.07

a: ICD-10 = 10th revision of the International Statistical Classification of Diseases and Related Health Problems

For peer review only

For peer review only - http://bmjopen.bmj.com/site/about/guidelines.xhtml

Table 3: Poisson regression for cancer-specific mortality and all-cancer mortality among Moluccan-Dutch, adjusted for age, gender and socioeconomic status.

Cancer type		Both genders		Males		Females
	N	RR[a] (95% CI[b])	N	RR (95% CI)	N	RR (95% CI)
Stomach	33	0.71 (0.50 – 1.00)	17	0.57 (0.35 – 0.91)	16	0.96 (0.59 – 1.57)
Liver	53	2.60 (1.98 – 3.42)	33	2.58 (1.83 – 3.64)	20	2.65 (1.69 – 4.17)
Cervix	15	-	0	-	15	1.89 (1.13 – 3.14)
Colorectal	138	0.95 (0.80 – 1.12)	81	1.01 (0.81 – 1.26)	57	0.87 (0.67 – 1.13)
Breast	134	-	-	-	134	1.08 (0.91 – 1.29)
Prostate	79	-	79	1.21 (0.97 – 1.51)	0	-
Bladder	27	0.75 (0.51 – 1.10)	16	0.64 (0.39 – 1.05)	11	1.03 (0.55 – 1.91)
Lung and lower airway	336	0.95 (0.85 – 1.05)	201	0.90 (0.78 – 1.03)	135	1.00 (0.84 – 1.19)
Esophagus	25	0.46 (0.31 – 0.68)	23	0.55 (0.37 – 0.83)	-	0.15 (0.04 – 0.62)
Corpus uteri	22	-	0	-	22	1.72 (1.12 – 2.64)
Ovaries	40	-	0	-	40	1.19 (0.87 – 1.63)
Pancreas	62	0.86 (0.67 – 1.11)	24	0.63 (0.42 – 0.95)	38	0.13 (0.82 – 1.55)
Kidney	20	0.61 (0.39 – 0.96)	14	0.70 (0.41 – 1.18)	-	0.46 (0.19 – 1.11)
Leukemia	48	1.21 (0.89 – 1.65)	25	1.17 (0.77 – 1.76)	23	1.27 (0.80 – 2.02)
Non-Hodgkin lymphoma	48	1.29 (0.96 – 1.73)	35	1.60 (1.13 – 2.27)	13	0.84 (0.48 – 1.49)
Nervous system	19	0.32 (0.18 – 0.58)	11	0.30 (0.13 – 0.66)	-	0.36 (0.15 – 0.86)
All cancers (ICD-10[c] codes C00 – D48)		0.98 (0.93 – 1.03)		0.92 (0.85 – 0.99)		1.05 (0.97 – 1.13)

532 a: RR = relative risk; b: CI = confidence interval; c: ICD-10 = 10th revision of the International Statistical Classification of Diseases and Related Health
533 Problems
534
535
536
537
538
539
540
541
542
543
544

For peer review only

Table 4: Poisson regression for cancer mortality among Moluccan-Dutch stratified by generation, adjusted for age, gender and socioeconomic status

Cancer type	Generation					
		1 (adult)		1 (minor)		2
	N	RR[a] (95% CI[b])	N	RR (95% CI)	N	RR (95% CI)
Stomach	10	0.48 (0.26 – 0.89)	10	0.65 (0.35 – 1.21)	13	1.27 (0.73 – 2.19)
Liver	29	3.96 (2.74 – 5.70)	14	1.79 (1.06 – 3.03)	-	1.86 (0.96 – 3.59)
Cervix	-	1.72 (0.55 – 5.34)	-	0.91 (0.23 – 3.64)	10	2.39 (1.28 – 4.47)
Colorectal	67	1.06 (0.83 – 1.34)	48	0.92 (0.69 – 1.22)	23	0.75 (0.50 – 1.13)
Breast	24	0.79 (0.53 – 1.17)	42	1.05 (0.77 – 1.42)	65	1.29 (1.01 – 1.65)
Prostate	71	1.61 (1.28 – 2.03)	-	0.45 (0.22 – 0.90)	-	-
Bladder	11	0.58 (0.32 – 1.05)	-	0.48 (0.20 – 1.16)	10	1.81 (0.97 – 3.38)
Lung and lower airway	99	0.82 (0.68 – 1.01)	99	0.70 (0.58 – 0.85)	135	1.42 (1.20 – 1.69)
Esophagus	-	0.37 (0.17 – 0.82)	-	0.39 (0.21 – 0.76)	10	0.63 (0.34 – 1.16)
Corpus uteri	-	1.21 (0.54 – 2.69)	-	1.52 (0.71 – 3.17)	-	3.14 (1.56 – 6.33)
Ovaries	-	0.67 (0.32 – 1.40)	15	1.18 (0.71 – 1.96)	17	1.76 (1.09 – 2.83)
Pancreas	29	1.09 (0.76 – 1.57)	23	0.81 (0.54 – 1.21)	10	0.59 (0.31 – 1.09)
Kidney	-	0.52 (0.23 – 1.15)	-	0.43 (0.18 – 1.04)	-	1.01 (0.50 – 2.02)
Leukemia	17	1.14 (0.71 – 1.84)	-	0.79 (0.41 – 1.51)	15	1.95 (1.17 – 3.25)
Non-Hodgkin lymphoma	24	1.68 (1.13 – 2.51)	12	0.98 (0.55 – 1.72)	-	1.03 (0.51 – 2.07)
Nervous system	-	0.36 (0.09 – 1.44)	-	0.39 (0.16 – 0.93)	-	0.25 (0.09 – 0.66)

a: RR = relative risk; b: CI = confidence interval

For peer review only

For peer review only - http://bmjopen.bmj.com/site/about/guidelines.xhtml

BMJ Open

A population-based retrospective study investigating cancer mortality among Moluccan migrants in the Netherlands: equal risk 65 years after immigration?

Journal:	*BMJ Open*
Manuscript ID	bmjopen-2019-029288.R1
Article Type:	Research
Date Submitted by the Author:	11-Jun-2019
Complete List of Authors:	van der Wal, Junus; Amsterdam UMC, location AMC, Department of Public Health<br>Bodewes, Adee; Amsterdam UMC, location AMC, Department of Public Health<br>Agyemang, Charles; Amsterdam UMC, location AMC, Department of Public Health<br>Kunst, Anton; Amsterdam UMC, location AMC, Department of Public Health
**Primary Subject Heading**:	Epidemiology
Secondary Subject Heading:	Oncology, Public health
Keywords:	cancer, mortality, ethnicity, Epidemiology < ONCOLOGY, the netherlands, moluccans

SCHOLARONE™ Manuscripts

A population-based retrospective study investigating cancer mortality among Moluccan migrants in the Netherlands: equal risk 65 years after immigration?

Junus M. van der Wal, Adee. J. Bodewes, Charles Agyemang, Anton E. Kunst

Authors affiliation: Amsterdam UMC, University of Amsterdam, Department of Public Health, Meibergdreef 9, 1105 AZ Amsterdam, Netherlands

Corresponding author: Junus M. van der Wal (j.m.vanderwal@amc.uva.nl / +31641889849)

Word count: 3011

Key words: cancer, mortality, ethnicity, the Netherlands, Moluccans, generation

Author contribution

JW: contributed to the design of the study, analyzed the data presented in this article and wrote the first draft of the article.

AB: contributed to the design of the study, lead the acquisition of the data, assisted in the analysis of the data and was involved in drafting the manuscript.

CA: contributed substantially to the drafting of the manuscript.

AK: contributed to the design of the study, assisted in the analysis of the data and commented on previous drafts of the manuscript.

For peer review only - http://bmjopen.bmj.com/site/about/guidelines.xhtml

Funding

This work was supported by the Dutch organization BUAT (platform for Moluccans) in order to obtain data from the Dutch national cause of death registry, Grant Number CJ2081008.

Competing interest

The authors declare that they have no conflict of interest.

Ethical approval:

According to the Medical Ethics Review Committee (METC) of the Amsterdam Medical Centre, an official approval of this study was not required because the act on Medical Research Involving Human Subjects (WMO) does not apply to this study (METC correspondence number W15-201#15.0240). The data was analyzed anonymously at Statistics Netherlands (CBS) computers, where all possible measures were taken to protect the privacy of subjects.

Availability of data and materials:

The data that support the findings of this study are available from CBS but restrictions apply to the availability of these data, which were used under license for the current study, and so are not publicly available.

Acknowledgments

We thank the Dutch organization BUAT (Platform for Moluccans) for contributing financially to the acquisition of data from the Dutch national cause of death registry. Furthermore, we thank CBS for their aid in obtaining the mortality and demographic data.

For peer review only

Abstract

Objective – To test the hypothesis that cancer mortality rates among the Moluccan-Dutch, the oldest non-Western migrant group to arrive in the Netherlands after the Second World War, are similar to those in the general Dutch population.

Design – Population-based retrospective study.

Setting – Data from the national cause of death registry in the Netherlands and municipal registries.

Participants – Using historic records containing family names of all Moluccan-Dutch who arrived in the Netherlands in 1951, we identified 35 581 Moluccan-Dutch persons in the national cause of death registry of the Netherlands. The reference group consisted of 15 866 538 persons of the general Dutch population.

Outcome measures – Mortality data were linked to demographic data from municipal registries. We calculated all-cancer and cancer-specific mortality and measured differences between the two groups using Poisson regression, adjusting for sex, age and area socioeconomic status. We conducted a sub-analysis for the first and second generation Moluccan-Dutch.

Results – There was no difference in all-cancer mortality between Moluccan-Dutch and the general Dutch population. Mortality was higher among Moluccan-Dutch for liver, cervix and corpus uteri cancers, but lower for stomach, esophagus, kidney and nervous system cancers. For most cancers, mortality risk as compared to the general Dutch population varied between different generations of Moluccan-Dutch.

Conclusions – Several decades after migration, the Moluccan-Dutch show similar all-cancer mortality, but different cancer-specific mortality rates, when compared to the general Dutch population.

For peer review only - http://bmjopen.bmj.com/site/about/guidelines.xhtml

Article summary

Strengths and limitations of this study

- This is the first study to investigate cancer mortality among the Moluccan-Dutch, the oldest non-western migrant group to enter the Netherlands after the second world war.
- We employed a novel approach to select persons from Moluccan descent from the Dutch national cause-of-death registry, by selecting persons based on the Moluccan family names registered in historical immigration documents, which were made available to us for research purposes.
- A limitation of this novel selection method is the fact the we were unable to identify second generation Moluccan-Dutch persons with a native Dutch father and consequently Dutch family name.
- Another limitation is the low number of deaths for most cancer types in the Moluccan-Dutch group, limiting statistical power.

For peer review only - http://bmjopen.bmj.com/site/about/guidelines.xhtml

For peer review only

Introduction

Ethnic disparities in the prevalence and mortality burden of different cancer types are known to exist in Europe.[1] For instance, migrants from low- and middle-income countries (LMIC) tend to show lower all-cancer mortality and morbidity. Moreover, cancer burden among LMIC-migrants is thought to be influenced by differences in cancer epidemiology between high-income countries (HICs) and LMIC and exposure to concordant risk factors.[2] When compared to their host population, cancers related to infection, such as liver, stomach and cervical cancer, are more common in migrants from LMICs; cancers considered to be related to Western lifestyle such as colorectal, breast and esophageal cancer are less common in these groups.[1]

Increasing evidence suggests that such epidemiological differences become less pronounced as LMIC-migrants reside in their destination country for a longer period, as local (Western) cancer-related risk factors will eventually predominate over risk factors associated with their country of origin.[2] Moreover, second generation migrants who were born in HICs lack early life risk factor exposures experienced by the first generation, such as possible higher infection pressure.[3] Accordingly, some studies on LMIC-migrants who have resided in their host country for more than one generation have suggested a shift in cancer epidemiology towards rates of the native population of their destination country.[4–6] In the Netherlands, such a pattern has been shown most consistent in Surinamese migrants for mortality caused by most major cancers, but is also suggested for migrants from Turkey, Morocco and the Netherland Antilles. [4,6] Given the limited number of studies on this topic, more research is necessary to further characterize the cancer risk profile in these groups and address the question to what extent ethnic disparities in cancer mortality persist or start to converge to those of the host population.

For this reason, we set out to assess the difference in cancer mortality between the general Dutch population and the Moluccan-Dutch, the oldest LMIC migrant group to settle in the Netherlands after the Second World War. The Moluccan-Dutch are a migrant group consisting of the former soldiers from

For peer review only - http://bmjopen.bmj.com/site/about/guidelines.xhtml

the Royal Netherlands East Indies Army (KNIL), the military force of the Netherlands in their former colony. These soldiers, and their families, were transported to the Netherlands in spring 1951 after decolonization of Indonesia. Even though their stay was supposed to be temporary, the majority settled in the Netherlands after their struggle to found their own republic on the Moluccan Islands failed. No considerable chain- or remigration occurred, resulting in a closed minority group, the vast majority of which resided in segregated Moluccan districts throughout the Netherlands.[7]

Earlier studies found differences in cancer standard incidence ratios and mortality between migrants from Indonesia and the general Dutch population.[8–10] A separate analysis of the Moluccan-Dutch is desirable, since their particular migration history resulted in a homogenous group within the Indonesian community with their own cultural practices, lifestyle and migration history, all of which can have a profound impact on cancer risk.

The aim of this study was to assess the difference in all cancer and cancer specific mortality rates between the Moluccan-Dutch and the general Dutch population, with a sub-analysis for the first and second generation. Since the Moluccan-Dutch have resided in the Netherlands for over 65 years, we expect local environmental factors in the Netherlands to reflect on mortality rates, **for instance** in cancers that are known to be influenced by factors such as lifestyle **or** exposure to infectious agents. Therefore, we hypothesize cancer mortality rates to be similar to those of the host population, especially for the second generation.[4,6]

For peer review only - http://bmjopen.bmj.com/site/about/guidelines.xhtml

Methods

Data sources and handling

For this study we used mortality data as recorded in the nationwide cause of death registry of Statistics Netherlands (CBS) between the years 2000 and 2013. Deaths in the Netherlands are documented by a physician on a death certificate form and cause of death is classified in accordance with the Tenth Revision of the International Classification of Diseases (ICD-10), after which it is recorded in the cause of death registry. Demographic data was derived from municipal registries and included date of birth, sex and place of residence. Both registries include all legal residents in the Netherlands, and are linked through personal identification numbers.

Moluccan-Dutch persons were identified in the registries based on surname. Using passenger lists of the various boats that transported Moluccans to the Netherlands in 1951, the Museum Maluku Netherlands created a list containing approximately 1700 surnames of the arriving Moluccan families. This list was used to identify and link persons of Moluccan descent in these registries. After identification and linking, data were anonymized. The reference group, referred to as 'general Dutch population' consisted of 15 866 538 persons with Dutch nationality.

We used birth year to determine Moluccan generations. We considered people born up to 1951, the year of migration, to be of the first generation and people born after 1951 but before 1970 of the second generation. We chose 1970 as cutoff point and excluded persons born hereafter, since Moluccan-Dutch born after this year will increasingly be offspring of the second generation (i.e. the third generation) and because the number of death in this group was low due to relatively young age. Furthermore, we created two groups within the first generation, based on whether the person was an adult (born before 1934) or a minor (born between 1934 and 1951) in the year that the Moluccan migrants were transported to the Netherlands. We made this stratification, since Moluccan-Dutch who migrated to the Netherlands as minor are expected to show distinctly different cancer risk exposures

For peer review only

For peer review only - http://bmjopen.bmj.com/site/about/guidelines.xhtml

than both the first generation adults, who have similar early life exposures from Indonesia but are less integrated in Dutch society, or the second generation, who lack early life exposures from Indonesia altogether.

In one analysis, we only included Moluccan-Dutch of the first and second generations, and we compared them to their Dutch counterparts born in the same periods. This reference group included 9 064 169 persons with Dutch nationality born up to 1970.

Area-level social economic status (SES) was used as proxy for individual socioeconomic status. Area-level SES was based on decile scores calculated by the Netherlands Institute for Social Research (SCP), reflecting SES of inhabitants of specific postal codes. A higher decile score indicates a higher area SES.

Data analysis

All-cancer mortality encompassed codes C00-D48 in the 10th revision of the International Statistical Classification of Diseases and Related Health Problems (ICD-10). Furthermore, we chose to investigate the most common types of cancer in both HICs and LMICs, as identified by the global cancer research project GLOBOCAN 2012.[11] In breast cancer, we only included female patients due to low number of deaths among males for this cancer type.

We present both absolute numbers of deaths, as well as age-adjusted death rates, using the direct method of standardization. In accordance with CBS guidelines, number of deaths lower than ten are not shown. Poisson regression analysis was used to investigate the size of difference in mortality attributable to the different types of cancer between Moluccan-Dutch and the general Dutch population, adjusted for sex, age-at-baseline and SES. Furthermore, we performed separate Poisson regression analysis for the first and second generation Moluccan-Dutch and for each sex. Based on these regression analysis, we present relative risks (RR) with 95% confidence intervals (CI). Analyses were performed using IBM SPSS Statistics 20.

For peer review only - http://bmjopen.bmj.com/site/about/guidelines.xhtml

Patient and public involvement

We used data from existing databases. No patients were involved the development of the research question, design of the study and sourcing of the data. Results of this study will be disseminated among the Moluccan-Dutch communities in the Netherlands via social media outlets.

For peer review only

For peer review only - http://bmjopen.bmj.com/site/about/guidelines.xhtml

Results

Demographic characteristics of our study population are presented in table 1. A total of 81 591 Moluccan-Dutch persons were included in this study. Within the Moluccan-Dutch group, 15.7% was of the first generation (5.7% migrated as adult and 10.0% as minor), 27.9% was of the second generation and 56.4% was of the second or third generation. The Moluccan-Dutch in our study population showed higher rates in the lower SES groups, when compared to the general Dutch population.

Table 2 gives overview of the investigated cancer types and the corresponding total number of deaths and age-adjusted death rates, as well as all-cancer mortality. In both the Moluccan-Dutch and the general Dutch population group, highest age-adjusted death rates were found for lung cancer, followed by colorectal and breast cancer, respectively.

Relative risk (RR) of cancer mortality for each investigated cancer type is presented in table 3. The Moluccan-Dutch showed similar all-cancer mortality risk when compared to the general Dutch population (RR = 0.98, CI: 0.93 – 1.03). However, Moluccan-Dutch men showed lower all-cancer mortality than men in the general Dutch population (RR = 0.92, CI: 0.85 – 0.99). Furthermore, the Moluccan-Dutch showed lower relative risk for mortality caused by cancer of the stomach (RR = 0.71, CI: 0.50-1.00), esophagus (RR = 0.46, CI: 0.31 – 0.68), kidneys (RR = 0.61, CI: 0.39 – 0.96) and nervous system (RR = 0.32, CI: 0.18 – 0.58), when compared to the general Dutch population. In contrast, they showed higher relative risk for mortality caused by cancer of the liver (RR = 2.60 CI: 1.98 – 3.42), cervix (RR = 1.89 CI: 1.13 – 3.14) and corpus uteri (RR = 1.72, CI: 1.12 – 2.64). Additionally, only Moluccan-Dutch men showed lower mortality risk for cancer of the stomach (RR = 0.57, CI: 0.35 – 0.91) and pancreas (RR = 0.63, CI: 0.42 – 0.95) and higher mortality risk for non-Hodgkin lymphoma (NHL) (RR = 1.60, CI: 1.13 – 2.27), when compared to men in the general Dutch population.

For peer review only - http://bmjopen.bmj.com/site/about/guidelines.xhtml

A regression analysis per generation within the Moluccan-Dutch group is presented in table 4. Among the first-generation adults, stomach cancer mortality was lower (RR = 0.48. CI: 0.26 – 0.89), but NHL mortality was higher (RR = 1.68, CI: 1.13 – 2.51), when compared to the general Dutch population. Furthermore, esophageal cancer mortality was lower in the first generation adults (RR = 0.37, CI: 0.17 – 0.82) and minors (RR = 0.39, CI: 0.21 – 0.76), whereas liver cancer mortality was higher among the first generation adults (RR = 3.96, CI: 2.74 – 5.70) and minors (RR = 1.79, CI: 1.06 – 3.03), when compared to their counterparts in the general Dutch population. Significantly elevated among the second generation, but not among first generation, were cancer of the cervix (RR = 2.39, CI: 1.28 – 4.47), breast (RR = 1.29, CI: 1.01 – 1.65), corpus uteri (RR = 3.14, CI: 1.56 – 6.33) and ovaries (RR = 1.76, CI: 1.09 – 2.83) and leukemia (RR = 1.95, CI: 1.17 – 3.25). Cancer of the brain and nervous system carried lower mortality among both first-generation minors (RR = 0.39, CI: 0.16 – 0.93) and the second generation Moluccans (RR = 0.25, CI: 0.09 – 0.66), when compared to the general Dutch population.

Two cancer types show inverse outcomes when comparing mortality risk of two consecutive generations of Moluccan-Dutch with the general Dutch population. Prostate cancer mortality was higher among first generation adults in the Moluccan-Dutch group when compared to their counterparts in the general Dutch population (RR = 1.61, CI: 1.28 – 2.03), but lower among first generation minors (RR = 0.45, CI: 0.22 – 0.90). Additionally, lower risk of dying of lung cancer was observed among first generation minors when compared to the general Dutch population (RR = 0.70, CI: 0.58 – 0.85), but the second-generation Moluccan-Dutch showed a higher risk (RR = 1.42, CI: 1.20 – 1.69).

For peer review only

For peer review only - http://bmjopen.bmj.com/site/about/guidelines.xhtml

Discussion

Key findings

Our objective was to compare cancer mortality between the Moluccan-Dutch and the general Dutch population. We found similar all-cancer mortality, but several differences in cancer-specific mortality across both the first and second generation Moluccan-Dutch.

Strengths and limitations

This is the first study that investigates cancer mortality specifically among the Moluccan-Dutch, by using historic records on the migration of the Moluccan migrants to the Netherlands, enabling us to select participant based on family name. One limitation of this study is the fact that, for most cancer types, the total number of deaths was small among the Moluccan-Dutch, limiting our ability to detect significant differences between Moluccan-Dutch and the general Dutch population. For the same reason, we had to exclude a separate analysis of third generation Moluccans.

Another limitation is that we were unable to include second generation Moluccan-Dutch persons with a Dutch father and consequently Dutch family name. It is estimated that approximately 75% of second generation Moluccan-Dutch have mixed parents.[12] It is uncertain to what extent our results are representative of all mixed-origin residents. Given that the influence of Moluccan culture often persists in families with one non-Moluccan parent, especially since approximately 40% of mixed-origin residents still live in segregated Moluccan districts, our results may apply, although in attenuated form, to these persons.[12]

Discussion of the key findings

As cancer risk depend not only on both factors associated with a migrant's country of origin (e.g. high infection pressure) but also on factors associated with the host country (e.g. Western lifestyle), the position of migrant within the host country is an important consideration when interpreting the

For peer review only - http://bmjopen.bmj.com/site/about/guidelines.xhtml

results. Moluccan-Dutch generally have lower-ranking occupations and are lower educated compared to native Dutch.[13] Since we were able to correct for area-level SES in our regression analyses, and this had limited impact on our results, we don't expect socioeconomic differences to have major impact on our results. Another important aspect is migrants' access to health care, as detection of cancer in later stages may increase mortality.[14] The evidence on this is equivocal, as Moluccan-Dutch are thought to show lower utilization of services by general practitioners, yet have similar number of visits to medical specialists, as compared to native Dutch.[15,16]

In the Netherlands, there is one earlier study reporting on cancer mortality among first and second generation Indonesian immigrants, which includes persons from the Moluccan Islands.[17] There seem to be multiple similarities in cancer mortality disparities between Moluccan-Dutch and the Indonesian immigrant group as a whole in the Netherlands, when compared to their Dutch counterparts. Both groups showed lower stomach cancer mortality, similar breast cancer mortality and higher liver and uterine cancer mortality when compared to their Dutch counterparts. However, the Indonesian immigrant group as a whole showed lower lung and colorectal cancer mortality when compared to native Dutch, whereas we found no differences between Moluccan-Dutch and the general Dutch population for these cancer types.

In Europe, a marked feature of cancer epidemiology in LMIC migrant groups is their lower all-cancer mortality risk.[1] However, we found that the Moluccan-Dutch experience similar all-cancer mortality when compared to the general Dutch population. Even though men showed significantly lower all-cancer mortality when compared to men in the general Dutch population, the difference was small as compared to other LMIC migrants in the Netherlands.[4,6]

Another characteristic of cancer epidemiology in LMIC migrants in Europe is a higher prevalence of cancer related to infection (pathogen-driven) and a lower prevalence of cancers thought to be related

For peer review only - http://bmjopen.bmj.com/site/about/guidelines.xhtml

to Western lifestyle (non-pathogen driven).[1] Our results imply that the Moluccan-Dutch do not fully adhere to this pattern. We will discuss all cancers for which we found a significant difference between Moluccan-Dutch and the general Dutch population and the cancer types that carried the highest mortality rates among both groups.

For cancers in which exposure to infectious agents is an important risk factor, we found higher mortality risk among Moluccan-Dutch for liver and cervical cancer and a lower mortality risk for stomach cancer, when compared to the general Dutch population.

- The higher liver cancer mortality among the Moluccan-Dutch, especially in the first

generation, might be reflective of early life exposure in Indonesia to higher burden of hepatitis B and C infection, the most important risk factor for liver cancer.[18] The fact that first generation migrants from Indonesia in the Netherlands show higher infectious hepatitis mortality than the general Dutch population further supports to this theory.[17] Another important risk factor for liver cancer is alcohol consumption. However, since a health survey among Moluccan-Dutch found their level of alcohol consumption to be lower when compared to native Dutch, this risk factor is unlikely to explain the difference found.[19]

- Cervical cancer mortality was only significantly elevated among Moluccan-Dutch women of the

second generation, when compared to their counterparts in the general Dutch population. This is in contrast with earlier research on LMIC-migrants in Europe where women of the first generation were at increased risk.[1,5] There is no data on the prevalence among Moluccan-Dutch women of the most important etiological factors for cervical cancer, specific strains of the sexually transmitted human papillomavirus.[20]

- The lower stomach cancer mortality among Moluccan-Dutch was mostly attributable to a

significantly lower risk in first generation adults when compared to the general Dutch population. Lower prevalence in Indonesia of the main risk factor, colonization with the bacteria Helicobacter pylori, might have had a protective effect in this group.[21,22]

For peer review only - http://bmjopen.bmj.com/site/about/guidelines.xhtml

Furthermore, we found that the Moluccan-Dutch carry higher mortality risk for cancer of the corpus uteri, but lower mortality risk for esophageal and kidney cancer and cancers of the nervous system.

- For cancer of the corpus uteri, the higher mortality risk among Moluccan-Dutch women when compared to women from the general Dutch population is probably not explained by an important risk factor, obesity, since obesity rate was found to be similar between the two groups.[19,23] Another important risk factor is (cumulative) estrogen exposure, but data on this topic in Moluccan-Dutch women is lacking.[24]
- For esophageal cancer, important risk factors are mostly related to Western lifestyle, such as smoking, alcohol consumption and obesity.[25] The Moluccan-Dutch show no significant difference in smoking or body mass index when compared to native Dutch, but they are thought to consume less alcohol, which could contribute to the results.[19]
- Kidney cancer mortality risk was lower among Moluccan-Dutch, when compared to the general Dutch population. A known risk factor for kidney cancer is hypertension, which actually may be more prevalent among Moluccan-Dutch.[15,19] Other risk factors are mostly related to a Western lifestyle, such as smoking and obesity, but prevalence of these particular risk factors is thought to be similar between Moluccan-Dutch and native Dutch.[19,26]
- Cancer of the nervous system mortality risk was lower among Moluccan-Dutch than among the general Dutch population. To date, only a few factors are known to influence the incidence of gliomas, such as ionizing radiation exposure, history of atopic constitution and some genetic risk factors.[27,28] Whether these factors are able to explain the lower risk among the Moluccan-Dutch is unclear.

We found no difference in risk of mortality between the entire group of Moluccan-Dutch and the general Dutch population for the cancer types that carried the highest mortality rates.

- In lung cancer, Moluccan-Dutch of the second generation showed a higher risk of dying from lung

For peer review only - http://bmjopen.bmj.com/site/about/guidelines.xhtml

cancer, when compared to the general Dutch population. Smoking is the most important risk factor of lung cancer.[29] As stated earlier, a health survey among Moluccan-Dutch found no difference in smoking between Moluccan-Dutch and native Dutch, but no stratification by generation was made.[19]

- In breast cancer, Moluccan-Dutch women of the second generation experienced increased mortality risk, when compared to the general Dutch population. Since migrant women are thought to show lower attendance at the national breast cancer screening program, lower detection rate in the early stages of breast cancer, lowering the chance of curable treatment options and consequently increase mortality, might contribute to the results.[14,30] However, this raises the question why first generation Moluccan-Dutch women do not show higher mortality risk for breast cancer.
- For colorectal cancer, there was no difference in the mortality risk between Moluccan-Dutch and the general Dutch population, which is in contrast with most other LMIC migrant groups in Europe.[1] One could speculate that the similar mortality risk detected in our study might be reflective of adaptation to Western lifestyle among the Moluccan-Dutch since 1951.[31]

In conclusion, several decades after migration from Indonesia to the Netherlands, the Moluccan-Dutch showed similar all-cancer mortality when compared to the general Dutch population, but differences exist in cancer-specific mortality between the two groups, in both the first and second generation. In liver and stomach cancer, an increased period of exposure to local risk factors in the Netherlands might have led to a shift in mortality towards rates of the general Dutch population. However, for most other cancer types, such a tendency was not detected, unlike some other studies in the Netherlands that included second generation migrants.[4,6] These results highlight the need for research aimed at characterizing the cancer profile of LMIC migrants in high-income countries in order to aid tailored preventative and diagnostic efforts.

For peer review only - http://bmjopen.bmj.com/site/about/guidelines.xhtml

References

1. Arnold, M., Razum, O. & Coebergh, J. W. Cancer risk diversity in non-western migrants to Europe: An overview of the literature. *Eur. J. Cancer* **46**, 2647–2659 (2010).
2. Gushulak, B. D. & MacPherson, D. W. The basic principles of migration health: Population mobility and gaps in disease prevalence. *Emerg. Themes Epidemiol.* **3**, 1–11 (2006).
3. Spallek, J., Zeeb, H. & Razum, O. What do we have to know from migrant ' s past exposures to understand their health status ? A life course approach. *Emerg. Themes Epidemiol.* **8**, 1–8 (2011).
4. Stirbu, I. *et al.* Cancer mortality rates among first and second generation migrants in the Netherlands: Convergence toward the rates of the native Dutch population. *Int. J. Cancer* **119**, 2665–2672 (2006).
5. Beiki, O., Allebeck, P., Nordqvist, T. & Moradi, T. Cervical, endometrial and ovarian cancers among immigrants in Sweden: Importance of age at migration and duration of residence. *Eur. J. Cancer* **45**, 107–118 (2009).
6. Williams, G. *et al.* Cancer incidence and mortality of Surinamese migrants in the Netherlands: In-between Surinamese and Dutch levels? *Cancer Causes Control* **24**, 1375–1383 (2013).
7. van Amersfoort, H. The waxing and waning of a diaspora: Moluccans in the Netherlands, 1950-2002. *J. Ethn. Migr. Stud.* **30**, 151–174 (2004).
8. Arnold, M., Aarts, M. J., Van Der Aa, M., Visser, O. & Coebergh, J. W. Investigating cervical, oesophageal and colon cancer risk and survival among migrants in the Netherlands. *Eur. J. Public Health* **23**, 867–873 (2013).
9. Arnold, M. *et al.* Diverging breast and stomach cancer incidence and survival in migrants in the Netherlands, 1996-2009. *Acta Oncol. (Madr).* **52**, 1195–1201 (2013).
10. Visser, O. & van Leeuwen, F. E. Cancer risk in first generation migrants in North-Holland/Flevoland, The Netherlands, 1995-2004. *Eur. J. Cancer* **43**, 901–908 (2007).

For peer review only

For peer review only - http://bmjopen.bmj.com/site/about/guidelines.xhtml

11. Torre, L. A. *et al.* Global Cancer Statistics, 2012. *CA a cancer J. Clin.* **65**, 87–108 (2015).

12. Bodewes, A. J., Agyemang, C. & Kunst, A. E. All-cause mortality among three generations of Moluccans in the Netherlands. *Eur. J. Public Health* **29**, 463–467 (2018).

13. Bodewes, A. J. & Kunst, A. E. Involving hard-to-reach ethnic minorities in low-budget health research: Lessons from a health survey among Moluccans in the Netherlands. *BMC Res. Notes* **9**, 1–8 (2016).

14. Vermeer, B. & Van den Muijsenbergh, M. E. T. C. The attendance of migrant women at the national breast cancer screening in the Netherlands 1997-2008. *Eur. J. Cancer Prev.* **19**, 195–8 (2010).

15. De Back, T. R., Bodewes, A. J., Brewster, L. M. & Kunst, A. E. Cardiovascular health and related health care use of Moluccan-Dutch immigrants. *PLoS One* **10**, 1–11 (2015).

16. Verhagen, I., Ros, W. J. G., Steunenberg, B., Laan, W. & De Wit, N. J. Differences in health care utilisation between elderly from ethnic minorities and ethnic Dutch elderly. *Int. J. Equity Health* **13**, 1–7 (2014).

17. Ho, L., Bos, V. & Kunst, A. E. Differences in cause-of-death patterns between the native Dutch and persons of Indonesian descent in the Netherlands. *Am. J. Public Health* **97**, 1616–1618 (2007).

18. El-Serag, H. B. Epidemiology of viral hepatitis and hepatocellular carcinoma. *Gastroenterology* **142**, 1264–1273.e1 (2012).

19. Van Der Wal, J. M., Bodewes, A. J., Agyemang, C. O. & Kunst, A. E. Higher self-reported prevalence of hypertension among Moluccan-Dutch than among the general population of the Netherlands: Results from a cross-sectional survey. *BMC Public Health* **14**, (2014).

20. Schiffman, M., Castle, P., Jeronimo, J., Rodriguez, A. & Wacholder, S. Human papillomavirus and cervical cancer. *Lancet* **370**, 890–907 (2007).

21. Huang, H. *et al.* Estimation of Cancer Burden Attributable to Infection in Asia. *J. Epidemiol.* **25**, 626–638 (2015).

For peer review only - http://bmjopen.bmj.com/site/about/guidelines.xhtml

22. Blankenstein, M. Van *et al.* The prevalence of helicobacter pylori infection in the Netherlands. **48**, (2013).

23. Reeves, G. K. *et al.* Cancer incidence and mortality in relation to body mass index in the Million Women Study: Cohort study. *Br. Med. J.* **335**, 1134–1139 (2007).

24. Collaborative Group on Epidemiological Studies on Endmetrial Cancer. Endometrial cancer and oral contraceptives: An individual participant meta-analysis of 27 276 women with endometrial cancer from 36 epidemiological studies. *Lancet Oncol.* **16**, 1061–1070 (2015).

25. Thrift, A. P. The epidemic of oesophageal carcinoma: Where are we now? *Cancer Epidemiol.* **41**, 88–95 (2016).

26. Weikert, S. & Ljungberg, B. Contemporary epidemiology of renal cell carcinoma: Perspectives of primary prevention. *World J. Urol.* **28**, 247–252 (2010).

27. Ostrom, Q. T. *et al.* The epidemiology of glioma in adults: A state of the science review. *Neuro. Oncol.* **16**, 896–913 (2014).

28. Maile, E. J., Barnes, I., Finlayson, A. E., Sayeed, S. & Ali, R. Nervous system and intracranial tumour incidence by ethnicity in England, 2001-2007: A descriptive epidemiological study. *PLoS One* **11**, 2001–2007 (2016).

29. Alberg, A. J., Brock, M. V., Ford, J. G., Samet, J. M. & Spivack, S. D. Epidemiology of lung cancer: Diagnosis and management of lung cancer, 3rd ed: American college of chest physicians evidence-based clinical practice guidelines. *Chest* **143**, 1–29 (2013).

30. Islanders, P. *et al.* Differences in Breast Cancer Stage, Treatment, and Survival by Race and Ethnicity. **163**, 1–7 (2003).

31. Rasool, S., Kadla, S. A., Rasool, V. & Ganai, B. A. A comparative overview of general risk factors associated with the incidence of colorectal cancer. *Tumor Biol.* **34**, 2469–2476 (2013).

For peer review only

For peer review only - http://bmjopen.bmj.com/site/about/guidelines.xhtml

List of abbreviations

LMIC: low- and middle income countries

HIC: high-income countries

KNIL: Royal Netherlands East Indies Army

CBS: Statistics Netherlands

SES: social economic status

SCP: Netherlands Institute for Social Research

ICD-10: 10th revision of the International Statistical Classification of Diseases and Related Health Problems

CI: confidence interval

RR: relative risk

NHL: non-Hodgkin lymphoma

For peer review only

For peer review only - http://bmjopen.bmj.com/site/about/guidelines.xhtml

Table 1: Demographic characteristics the Moluccan-Dutch and the Dutch general population				
	Moluccan-Dutch		Dutch general population	
	N	%	N	%
Sex				
Male	40062	49.1	7917402	49.9
Female	41529	50.9	7949136	50.1
Generation (birth year)				
1 adult (<1934)	4642	5.7	2480434	15.6
1 minor (1935-1951)	8185	10.0	2639408	16.6
2 (1951-1969)	22754	27.9	3944327	24.9
≥2 (1970 >)	46010	56.4	6802369	42.9
Decile score				
1 (lowest SES[a])	7017	8.6	729861	4.6
2	7669	9.4	951992	6.0
3	10363	12.7	1729453	10.9
4	11341	13.9	1745319	11.0
5	11096	13.6	2491047	15.7
6	6935	8.5	1919851	12.1
7	6283	7.7	1856385	11.7
8	4732	5.8	1174124	7.4
9	7262	8.9	1586654	10.0
10 (highest SES)	8648	10.6	1650120	10.4
Missing	245	0.3	31732	0.2
Total	81591	100	15866538	100

a: SES = socioeconomic status

For peer review only

Table 2: Absolute numbers of death and age-standardized death rates per cancer type					
		Absolute numbers of death		Age-standardized death rates per 100,000	
Type of cancer	ICD-10[a] code	Dutch general population	Moluccan-Dutch	Dutch general population	Moluccan-Dutch
Stomach	C16	18443	33	4.98	3.51
Liver cell	C22	7227	53	2.03	6.37
Cervix uteri	C53	2488	15	0.77	1.51
Colorectal	C18-21	59548	138	16.11	15.51
Breast	C50	41324	134	12.59	13.20
Prostate	C61	30975	79	7.60	10.93
Bladder	C67	14875	27	3.86	2.91
Lung and lower airway	C33-34	119520	336	34.67	34.89
Esophagus	C15	18683	25	5.51	2.55
Corpus uteri	C54	5189	22	1.38	2.15
Ovaries	C56	12239	40	3.52	3.76
Pancreas	C25	27351	62	7.64	6.95
Kidney	C64	11491	20	3.24	1.98
Leukemia	C91-95	14349	48	3.71	5.04
Non-Hodgkin lymphoma	C82-85, C96	13620	48	3.70	5.13
Nervous-system	C70-72	10893	19	3.36	1.07

a: ICD-10 = 10th revision of the International Statistical Classification of Diseases and Related Health Problems

For peer review only

Table 3: Poisson regression for cancer-specific mortality and all-cancer mortality among Moluccan-Dutch, adjusted for age-at-baseline and socioeconomic status.

Cancer type	Both sexes		Males		Females	
	N	RR[a] (95% CI[b])	N	RR (95% CI)	N	RR (95% CI)
Stomach	33	0.71 (0.50 – 1.00)	17	0.57 (0.35 – 0.91)	16	0.96 (0.59 – 1.57)
Liver	53	2.60 (1.98 – 3.42)	33	2.58 (1.83 – 3.64)	20	2.65 (1.69 – 4.17)
Cervix	15	-	-	-	15	1.89 (1.13 – 3.14)
Colorectal	138	0.95 (0.80 – 1.12)	81	1.01 (0.81 – 1.26)	57	0.87 (0.67 – 1.13)
Breast	134	-	-	-	134	1.08 (0.91 – 1.29)
Prostate	79	-	79	1.21 (0.97 – 1.51)	-	-
Bladder	27	0.75 (0.51 – 1.10)	16	0.64 (0.39 – 1.05)	11	1.03 (0.55 – 1.91)
Lung and lower airway	336	0.95 (0.85 – 1.05)	201	0.90 (0.78 – 1.03)	135	1.00 (0.84 – 1.19)
Esophagus	25	0.46 (0.31 – 0.68)	23	0.55 (0.37 – 0.83)	<10	0.15 (0.04 – 0.62)
Corpus uteri	22	-	-	-	22	1.72 (1.12 – 2.64)
Ovaries	40	-	-	-	40	1.19 (0.87 – 1.63)
Pancreas	62	0.86 (0.67 – 1.11)	24	0.63 (0.42 – 0.95)	38	0.13 (0.82 – 1.55)
Kidney	20	0.61 (0.39 – 0.96)	14	0.70 (0.41 – 1.18)	<10	0.46 (0.19 – 1.11)
Leukemia	48	1.21 (0.89 – 1.65)	25	1.17 (0.77 – 1.76)	23	1.27 (0.80 – 2.02)
Non-Hodgkin lymphoma	48	1.29 (0.96 – 1.73)	35	1.60 (1.13 – 2.27)	13	0.84 (0.48 – 1.49)
Nervous system	19	0.32 (0.18 – 0.58)	11	0.30 (0.13 – 0.66)	<10	0.36 (0.15 – 0.86)
All cancers (ICD-10[d] codes C00 – D48)		0.98 (0.93 – 1.03)		0.92 (0.85 – 0.99)		1.05 (0.97 – 1.13)

a: RR = relative risk; b: CI = confidence interval; c: ICD-10 = 10th revision of the International Statistical Classification of Diseases and Related Health Problems. N < 10 is not shown in accordance with Statistics Netherlands (CBS) guidelines.

For peer review only

Table 4: Poisson regression for cancer mortality among Moluccan-Dutch stratified by generation, adjusted for age-at-baseline, sex and socioeconomic status						
Cancer type	Generation					
		1 (adult)		1 (minor)		2
	N	RR[a] (95% CI[b])	N	RR (95% CI)	N	RR (95% CI)
Stomach	10	0.48 (0.26 – 0.89)	10	0.65 (0.35 – 1.21)	13	1.27 (0.73 – 2.19)
Liver	29	3.96 (2.74 – 5.70)	14	1.79 (1.06 – 3.03)	<10	1.86 (0.96 – 3.59)
Cervix	<10	1.72 (0.55 – 5.34)	<10	0.91 (0.23 – 3.64)	10	2.39 (1.28 – 4.47)
Colorectal	67	1.06 (0.83 – 1.34)	48	0.92 (0.69 – 1.22)	23	0.75 (0.50 – 1.13)
Breast	24	0.79 (0.53 – 1.17)	42	1.05 (0.77 – 1.42)	65	1.29 (1.01 – 1.65)
Prostate	71	1.61 (1.28 – 2.03)	<10	0.45 (0.22 – 0.90)	-	-
Bladder	11	0.58 (0.32 – 1.05)	<10	0.48 (0.20 – 1.16)	10	1.81 (0.97 – 3.38)
Lung and lower airway	99	0.82 (0.68 – 1.01)	99	0.70 (0.58 – 0.85)	135	1.42 (1.20 – 1.69)
Esophagus	<10	0.37 (0.17 – 0.82)	<10	0.39 (0.21 – 0.76)	10	0.63 (0.34 – 1.16)
Corpus uteri	<10	1.21 (0.54 – 2.69)	<10	1.52 (0.71 – 3.17)	<10	3.14 (1.56 – 6.33)
Ovaries	<10	0.67 (0.32 – 1.40)	15	1.18 (0.71 – 1.96)	17	1.76 (1.09 – 2.83)
Pancreas	29	1.09 (0.76 – 1.57)	23	0.81 (0.54 – 1.21)	10	0.59 (0.31 – 1.09)
Kidney	<10	0.52 (0.23 – 1.15)	<10	0.43 (0.18 – 1.04)	<10	1.01 (0.50 – 2.02)
Leukemia	17	1.14 (0.71 – 1.84)	<10	0.79 (0.41 – 1.51)	15	1.95 (1.17 – 3.25)
Non-Hodgkin lymphoma	24	1.68 (1.13 – 2.51)	12	0.98 (0.55 – 1.72)	<10	1.03 (0.51 – 2.07)
Nervous system	<10	0.36 (0.09 – 1.44)	<10	0.39 (0.16 – 0.93)	<10	0.25 (0.09 – 0.66)

a: RR = relative risk; b: CI = confidence interval. N < 10 is not shown in accordance with Statistics Netherlands (CBS) guidelines.

For peer review only - http://bmjopen.bmj.com/site/about/guidelines.xhtml

Reporting checklist for cohort study.

Based on the STROBE cohort guidelines.

Instructions to authors

Complete this checklist by entering the page numbers from your manuscript where readers will find each of the items listed below.

Your article may not currently address all the items on the checklist. Please modify your text to include the missing information. If you are certain that an item does not apply, please write "n/a" and provide a short explanation.

Upload your completed checklist as an extra file when you submit to a journal.

In your methods section, say that you used the STROBE cohort reporting guidelines, and cite them as:

von Elm E, Altman DG, Egger M, Pocock SJ, Gotzsche PC, Vandenbroucke JP. The Strengthening the Reporting of Observational Studies in Epidemiology (STROBE) Statement: guidelines for reporting observational studies.

		Reporting Item	Page Number
Title	#1a	Indicate the study's design with a commonly used term in the title or the abstract	1
Abstract	#1b	Provide in the abstract an informative and balanced summary of what was done and what was found	3

For peer review only

Background / rationale	#2	Explain the scientific background and rationale for the investigation being reported	5-6
Objectives	#3	State specific objectives, including any prespecified hypotheses	6
Study design	#4	Present key elements of study design early in the paper	7-8
Setting	#5	Describe the setting, locations, and relevant dates, including periods of recruitment, exposure, follow-up, and data collection	7-8
Eligibility criteria	#6a	Give the eligibility criteria, and the sources and methods of selection of participants. Describe methods of follow-up.	7-8
	#6b	For matched studies, give matching criteria and number of exposed and unexposed	n/a
Variables	#7	Clearly define all outcomes, exposures, predictors, potential confounders, and effect modifiers. Give diagnostic criteria, if applicable	7-8
Data sources / measurement	#8	For each variable of interest give sources of data and details of methods of assessment (measurement). Describe comparability of assessment methods if there is more than one group. Give information separately for for exposed and unexposed groups if applicable.	7-8
Bias	#9	Describe any efforts to address potential sources of bias	12
Study size	#10	Explain how the study size was arrived at	7-10
Quantitative	#11	Explain how quantitative variables were handled in the	7-8

For peer review only

variables		analyses. If applicable, describe which groupings were chosen, and why	
Statistical methods	#12a	Describe all statistical methods, including those used to control for confounding	7-8
	#12b	Describe any methods used to examine subgroups and interactions	7-8
	#12c	Explain how missing data were addressed	n/a
	#12d	If applicable, explain how loss to follow-up was addressed	n/a
	#12e	Describe any sensitivity analyses	n/a
Participants	#13a	Report numbers of individuals at each stage of study—eg numbers potentially eligible, examined for eligibility, confirmed eligible, included in the study, completing follow-up, and analysed. Give information separately for for exposed and unexposed groups if applicable.	7-8, 10
	#13b	Give reasons for non-participation at each stage	n/a
	#13c	Consider use of a flow diagram	n/a
Descriptive data	#14a	Give characteristics of study participants (eg demographic, clinical, social) and information on exposures and potential confounders. Give information separately for exposed and unexposed groups if applicable.	10
	#14b	Indicate number of participants with missing data for each variable of interest	21

For peer review only

	#14c	Summarise follow-up time (eg, average and total amount)	n/a
Outcome data	#15	Report numbers of outcome events or summary measures over time. Give information separately for exposed and unexposed groups if applicable.	10-11
Main results	#16a	Give unadjusted estimates and, if applicable, confounder-adjusted estimates and their precision (eg, 95% confidence interval). Make clear which confounders were adjusted for and why they were included	10-11
	#16b	Report category boundaries when continuous variables were categorized	7-8
	#16c	If relevant, consider translating estimates of relative risk into absolute risk for a meaningful time period	n/a
Other analyses	#17	Report other analyses done—e.g., analyses of subgroups and interactions, and sensitivity analyses	10-11
Key results	#18	Summarise key results with reference to study objectives	12
Limitations	#19	Discuss limitations of the study, taking into account sources of potential bias or imprecision. Discuss both direction and magnitude of any potential bias.	12
Interpretation	#20	Give a cautious overall interpretation considering objectives, limitations, multiplicity of analyses, results from similar studies, and other relevant evidence.	12-16
Generalisability	#21	Discuss the generalisability (external validity) of the study	12, 16

For peer review only

		results	
Funding	#22	Give the source of funding and the role of the funders for the present study and, if applicable, for the original study on which the present article is based	2

The STROBE checklist is distributed under the terms of the Creative Commons Attribution License CC-BY. This checklist can be completed online using https://www.goodreports.org/, a tool made by the EQUATOR Network in collaboration with Penelope.ai

For peer review only

BMJ Open

A population-based retrospective study comparing cancer mortality between Moluccan migrants and the general Dutch population: equal risk 65 years after immigration?

Journal:	*BMJ Open*
Manuscript ID	bmjopen-2019-029288.R2
Article Type:	Research
Date Submitted by the Author:	23-Jul-2019
Complete List of Authors:	van der Wal, Junus; Amsterdam UMC, location AMC, Department of Public Health<br>Bodewes, Adee; Amsterdam UMC, location AMC, Department of Public Health<br>Agyemang, Charles; Amsterdam UMC, location AMC, Department of Public Health<br>Kunst, Anton; Amsterdam UMC, location AMC, Department of Public Health
**Primary Subject Heading**:	Epidemiology
Secondary Subject Heading:	Oncology, Public health
Keywords:	cancer, mortality, ethnicity, Epidemiology < ONCOLOGY, the netherlands, moluccans

SCHOLARONE™
Manuscripts

A population-based retrospective study comparing cancer mortality between Moluccan migrants and the general Dutch population: equal risk 65 years after immigration?

Junus M. van der Wal, Adee. J. Bodewes, Charles Agyemang, Anton E. Kunst

Authors affiliation: Amsterdam UMC, University of Amsterdam, Department of Public Health, Meibergdreef 9, 1105 AZ Amsterdam, Netherlands

Corresponding author: Junus M. van der Wal (j.m.vanderwal@amc.uva.nl / +31641889849)

Word count: 3011

Key words: cancer, mortality, ethnicity, the Netherlands, Moluccans, generation

Author contribution

JW: contributed to the design of the study, analyzed the data presented in this article and wrote the first draft of the article.

AB: contributed to the design of the study, lead the acquisition of the data, assisted in the analysis of the data and was involved in drafting the manuscript.

CA: contributed substantially to the drafting of the manuscript.

AK: contributed to the design of the study, assisted in the analysis of the data and commented on previous drafts of the manuscript.

For peer review only - http://bmjopen.bmj.com/site/about/guidelines.xhtml

Funding

This work was supported by the Dutch organization BUAT (platform for Moluccans) in order to obtain data from the Dutch national cause of death registry, Grant Number CJ2081008.

Competing interest

The authors declare that they have no conflict of interest.

Ethical approval:

According to the Medical Ethics Review Committee (METC) of the Amsterdam Medical Centre, an official approval of this study was not required because the act on Medical Research Involving Human Subjects (WMO) does not apply to this study (METC correspondence number W15-201#15.0240). The data was analyzed anonymously at Statistics Netherlands (CBS) computers, where all possible measures were taken to protect the privacy of subjects.

Availability of data and materials:

The data that support the findings of this study are available from CBS but restrictions apply to the availability of these data, which were used under license for the current study, and so are not publicly available.

Acknowledgments

We thank the Dutch organization BUAT (Platform for Moluccans) for contributing financially to the acquisition of data from the Dutch national cause of death registry. Furthermore, we thank CBS for their aid in obtaining the mortality and demographic data.

For peer review only

Abstract

Objective – To test the hypothesis that cancer mortality rates among the Moluccan-Dutch, the oldest non-Western migrant group to arrive in the Netherlands after the Second World War, are similar to those in the general Dutch population.

Design – Population-based retrospective study.

Setting – Data from the national cause of death registry in the Netherlands and municipal registries.

Participants – Using historic records containing family names of all Moluccan-Dutch who arrived in the Netherlands in 1951, we identified 35 581 Moluccan-Dutch persons in the national cause of death registry of the Netherlands. The reference group consisted of 15 866 538 persons of the general Dutch population.

Outcome measures – Mortality data were linked to demographic data from municipal registries. We calculated all-cancer and cancer-specific mortality and measured differences between the two groups using Poisson regression, adjusting for sex, age and area socioeconomic status. We conducted a sub-analysis for the first and second generation Moluccan-Dutch.

Results – There was no difference in all-cancer mortality between Moluccan-Dutch and the general Dutch population. Mortality was higher among Moluccan-Dutch for liver, cervix and corpus uteri cancers, but lower for stomach, esophagus, kidney and nervous system cancers. For most cancers, mortality risk as compared to the general Dutch population varied between different generations of Moluccan-Dutch.

Conclusions – Several decades after migration, the Moluccan-Dutch show similar all-cancer mortality, but different cancer-specific mortality rates, when compared to the general Dutch population.

For peer review only

For peer review only - http://bmjopen.bmj.com/site/about/guidelines.xhtml

Article summary

Strengths and limitations of this study

- This is the first study to investigate cancer mortality among the Moluccan-Dutch, the oldest non-western migrant group to enter the Netherlands after the second world war.
- We employed a novel approach to select persons from Moluccan descent from the Dutch national cause-of-death registry, by selecting persons based on the Moluccan family names registered in historical immigration documents, which were made available to us for research purposes.
- A limitation of this novel selection method is the fact the we were unable to identify second generation Moluccan-Dutch persons with a native Dutch father and consequently Dutch family name.
- Another limitation is the low number of deaths for most cancer types in the Moluccan-Dutch group, limiting statistical power.

For peer review only - http://bmjopen.bmj.com/site/about/guidelines.xhtml

Introduction

Ethnic disparities in the prevalence and mortality burden of different cancer types are known to exist in Europe.[1] For instance, migrants from low- and middle-income countries (LMIC) tend to show lower all-cancer mortality and morbidity. Moreover, cancer burden among LMIC-migrants is thought to be influenced by differences in cancer epidemiology between high-income countries (HICs) and LMIC and exposure to concordant risk factors.[2] When compared to their host population, cancers related to infection, such as liver, stomach and cervical cancer, are more common in migrants from LMICs; cancers considered to be related to Western lifestyle such as colorectal, breast and esophageal cancer are less common in these groups.[1]

Increasing evidence suggests that such epidemiological differences become less pronounced as LMIC-migrants reside in their destination country for a longer period, as local (Western) cancer-related risk factors will eventually predominate over risk factors associated with their country of origin.[2] Moreover, second generation migrants who were born in HICs lack early life risk factor exposures experienced by the first generation, such as possible higher infection pressure.[3] Accordingly, some studies on LMIC-migrants who have resided in their host country for more than one generation have suggested a shift in cancer epidemiology towards rates of the native population of their destination country.[4–6] In the Netherlands, such a pattern has been shown most consistent in Surinamese migrants for mortality caused by most major cancers, but is also suggested for migrants from Turkey, Morocco and the Netherland Antilles. [4,6] Given the limited number of studies on this topic, more research is necessary to further characterize the cancer risk profile in these groups and address the question to what extent ethnic disparities in cancer mortality persist or start to converge to those of the host population.

For this reason, we set out to assess the difference in cancer mortality between the general Dutch population and the Moluccan-Dutch, the oldest LMIC migrant group to settle in the Netherlands after the Second World War. The Moluccan-Dutch are a migrant group consisting of the former soldiers from

For peer review only - http://bmjopen.bmj.com/site/about/guidelines.xhtml

the Royal Netherlands East Indies Army (KNIL), the military force of the Netherlands in their former colony. These soldiers, and their families, were transported to the Netherlands in spring 1951 after decolonization of Indonesia. Even though their stay was supposed to be temporary, the majority settled in the Netherlands after their struggle to found their own republic on the Moluccan Islands failed. No considerable chain- or remigration occurred, resulting in a closed minority group, the vast majority of which resided in segregated Moluccan districts throughout the Netherlands.[7]

Earlier studies found differences in cancer standard incidence ratios and mortality between migrants from Indonesia and the general Dutch population.[8–10] A separate analysis of the Moluccan-Dutch is desirable, since their particular migration history resulted in a homogenous group within the Indonesian community with their own cultural practices, lifestyle and migration history, all of which can have a profound impact on cancer risk.

The aim of this study was to assess the difference in all cancer and cancer specific mortality rates between the Moluccan-Dutch and the general Dutch population, with a sub-analysis for the first and second generation. Since the Moluccan-Dutch have resided in the Netherlands for over 65 years, we expect local environmental factors in the Netherlands to reflect on mortality rates, **for instance** in cancers that are known to be influenced by factors such as lifestyle **or** exposure to infectious agents. Therefore, we hypothesize cancer mortality rates to be similar to those of the host population, especially for the second generation.[4,6]

For peer review only - http://bmjopen.bmj.com/site/about/guidelines.xhtml

Methods

Data sources and handling

For this study we used mortality data as recorded in the nationwide cause of death registry of Statistics Netherlands (CBS) between the years 2000 and 2013. Deaths in the Netherlands are documented by a physician on a death certificate form and cause of death is classified in accordance with the Tenth Revision of the International Classification of Diseases (ICD-10), after which it is recorded in the cause of death registry. Demographic data was derived from municipal registries and included date of birth, sex and place of residence. Both registries include all legal residents in the Netherlands, and are linked through personal identification numbers.

Moluccan-Dutch persons were identified in the registries based on surname. Using passenger lists of the various boats that transported Moluccans to the Netherlands in 1951, the Museum Maluku Netherlands created a list containing approximately 1700 surnames of the arriving Moluccan families. This list was used to identify and link persons of Moluccan descent in these registries. After identification and linking, data were anonymized. The reference group, referred to as 'general Dutch population' consisted of 15 866 538 persons with Dutch nationality.

For peer review only

We used birth year to determine Moluccan generations. We considered people born up to 1951, the year of migration, to be of the first generation and people born after 1951 but before 1970 of the second generation. We chose 1970 as cutoff point and excluded persons born hereafter, since Moluccan-Dutch born after this year will increasingly be offspring of the second generation (i.e. the third generation) and because the number of death in this group was low due to relatively young age. Furthermore, we created two groups within the first generation, based on whether the person was an adult (born before 1934) or a minor (born between 1934 and 1951) in the year that the Moluccan migrants were transported to the Netherlands. We made this stratification, since Moluccan-Dutch who migrated to the Netherlands as minor are expected to show distinctly different cancer risk exposures

For peer review only - http://bmjopen.bmj.com/site/about/guidelines.xhtml

than both the first generation adults, who have similar early life exposures from Indonesia but are less integrated in Dutch society, or the second generation, who lack early life exposures from Indonesia altogether.

In one analysis, we only included Moluccan-Dutch of the first and second generations, and we compared them to their Dutch counterparts born in the same periods. This reference group included 9 064 169 persons with Dutch nationality born up to 1970.

Area-level social economic status (SES) was used as proxy for individual socioeconomic status. Area-level SES was based on decile scores calculated by the Netherlands Institute for Social Research (SCP), reflecting SES of inhabitants of specific postal codes. A higher decile score indicates a higher area SES.

Data analysis

All-cancer mortality encompassed codes C00-D48 in the 10th revision of the International Statistical Classification of Diseases and Related Health Problems (ICD-10). Furthermore, we chose to investigate the most common types of cancer in both HICs and LMICs, as identified by the global cancer research project GLOBOCAN 2012.[11] In breast cancer, we only included female patients due to low number of deaths among males for this cancer type.

We present both absolute numbers of deaths, as well as age-adjusted death rates, using the direct method of standardization. In accordance with CBS guidelines, number of deaths lower than ten are not shown. Poisson regression analysis was used to investigate the size of difference in mortality attributable to the different types of cancer between Moluccan-Dutch and the general Dutch population, adjusted for sex, age-at-baseline (age in the year 2000, beginning of mortality data collection) and SES. Furthermore, we performed separate Poisson regression analysis for the first and second generation Moluccan-Dutch and for each sex. Based on these regression analysis, we present

For peer review only - http://bmjopen.bmj.com/site/about/guidelines.xhtml

relative risks (RR) with 95% confidence intervals (CI). Analyses were performed using IBM SPSS Statistics 20.

Patient and public involvement

We used data from existing databases. No patients were involved the development of the research question, design of the study and sourcing of the data. Results of this study will be disseminated among the Moluccan-Dutch communities in the Netherlands via social media outlets.

For peer review only

For peer review only - http://bmjopen.bmj.com/site/about/guidelines.xhtml

Results

Demographic characteristics of our study population are presented in table 1. A total of 81 591 Moluccan-Dutch persons were included in this study. Within the Moluccan-Dutch group, 15.7% was of the first generation (5.7% migrated as adult and 10.0% as minor), 27.9% was of the second generation and 56.4% was of the second or third generation. The Moluccan-Dutch in our study population showed higher rates in the lower SES groups, when compared to the general Dutch population.

Table 2 gives overview of the investigated cancer types and the corresponding total number of deaths and age-adjusted death rates, as well as all-cancer mortality. In both the Moluccan-Dutch and the general Dutch population group, highest age-adjusted death rates were found for lung cancer, followed by colorectal and breast cancer, respectively.

Relative risk (RR) of cancer mortality for each investigated cancer type is presented in table 3. The Moluccan-Dutch showed similar all-cancer mortality risk when compared to the general Dutch population (RR = 0.98, CI: 0.93 – 1.03). However, Moluccan-Dutch men showed lower all-cancer mortality than men in the general Dutch population (RR = 0.92, CI: 0.85 – 0.99). Furthermore, the Moluccan-Dutch showed lower relative risk for mortality caused by cancer of the stomach (RR = 0.71, CI: 0.50-1.00), esophagus (RR = 0.46, CI: 0.31 – 0.68), kidneys (RR = 0.61, CI: 0.39 – 0.96) and nervous system (RR = 0.32, CI: 0.18 – 0.58), when compared to the general Dutch population. In contrast, they showed higher relative risk for mortality caused by cancer of the liver (RR = 2.60 CI: 1.98 – 3.42), cervix (RR = 1.89 CI: 1.13 – 3.14) and corpus uteri (RR = 1.72, CI: 1.12 – 2.64). Additionally, only Moluccan-Dutch men showed lower mortality risk for cancer of the stomach (RR = 0.57, CI: 0.35 – 0.91) and pancreas (RR = 0.63, CI: 0.42 – 0.95) and higher mortality risk for non-Hodgkin lymphoma (NHL) (RR = 1.60, CI: 1.13 – 2.27), when compared to men in the general Dutch population.

For peer review only - http://bmjopen.bmj.com/site/about/guidelines.xhtml

A regression analysis per generation within the Moluccan-Dutch group is presented in table 4. Among the first-generation adults, stomach cancer mortality was lower (RR = 0.48. CI: 0.26 – 0.89), but NHL mortality was higher (RR = 1.68, CI: 1.13 – 2.51), when compared to the general Dutch population. Furthermore, esophageal cancer mortality was lower in the first generation adults (RR = 0.37, CI: 0.17 – 0.82) and minors (RR = 0.39, CI: 0.21 – 0.76), whereas liver cancer mortality was higher among the first generation adults (RR = 3.96, CI: 2.74 – 5.70) and minors (RR = 1.79, CI: 1.06 – 3.03), when compared to their counterparts in the general Dutch population. Significantly elevated among the second generation, but not among first generation, were cancer of the cervix (RR = 2.39, CI: 1.28 – 4.47), breast (RR = 1.29, CI: 1.01 – 1.65), corpus uteri (RR = 3.14, CI: 1.56 – 6.33) and ovaries (RR = 1.76, CI: 1.09 – 2.83) and leukemia (RR = 1.95, CI: 1.17 – 3.25). Cancer of the brain and nervous system carried lower mortality among both first-generation minors (RR = 0.39, CI: 0.16 – 0.93) and the second generation Moluccans (RR = 0.25, CI: 0.09 – 0.66), when compared to the general Dutch population.

Two cancer types show inverse outcomes when comparing mortality risk of two consecutive generations of Moluccan-Dutch with the general Dutch population. Prostate cancer mortality was higher among first generation adults in the Moluccan-Dutch group when compared to their counterparts in the general Dutch population (RR = 1.61, CI: 1.28 – 2.03), but lower among first generation minors (RR = 0.45, CI: 0.22 – 0.90). Additionally, lower risk of dying of lung cancer was observed among first generation minors when compared to the general Dutch population (RR = 0.70, CI: 0.58 – 0.85), but the second-generation Moluccan-Dutch showed a higher risk (RR = 1.42, CI: 1.20 – 1.69).

For peer review only

For peer review only - http://bmjopen.bmj.com/site/about/guidelines.xhtml

Discussion

Key findings

Our objective was to compare cancer mortality between the Moluccan-Dutch and the general Dutch population. We found similar all-cancer mortality, but several differences in cancer-specific mortality across both the first and second generation Moluccan-Dutch.

Strengths and limitations

This is the first study that investigates cancer mortality specifically among the Moluccan-Dutch, by using historic records on the migration of the Moluccan migrants to the Netherlands, enabling us to select participant based on family name. One limitation of this study is the fact that, for most cancer types, the total number of deaths was small among the Moluccan-Dutch, limiting our ability to detect significant differences between Moluccan-Dutch and the general Dutch population. For the same reason, we had to exclude a separate analysis of third generation Moluccans.

Another limitation is that we were unable to include second generation Moluccan-Dutch persons with a Dutch father and consequently Dutch family name. It is estimated that approximately 75% of second generation Moluccan-Dutch have mixed parents.[12] It is uncertain to what extent our results are representative of all mixed-origin residents. Given that the influence of Moluccan culture often persists in families with one non-Moluccan parent, especially since approximately 40% of mixed-origin residents still live in segregated Moluccan districts, our results may apply, although in attenuated form, to these persons.[12]

Discussion of the key findings

As cancer risk depend not only on both factors associated with a migrant's country of origin (e.g. high infection pressure) but also on factors associated with the host country (e.g. Western lifestyle), the position of migrant within the host country is an important consideration when interpreting the

For peer review only - http://bmjopen.bmj.com/site/about/guidelines.xhtml

results. Moluccan-Dutch generally have lower-ranking occupations and are lower educated compared to native Dutch.[13] Since we were able to correct for area-level SES in our regression analyses, and this had limited impact on our results, we don't expect socioeconomic differences to have major impact on our results. Another important aspect is migrants' access to health care, as detection of cancer in later stages may increase mortality.[14] The evidence on this is equivocal, as Moluccan-Dutch are thought to show lower utilization of services by general practitioners, yet have similar number of visits to medical specialists, as compared to native Dutch.[15,16]

In the Netherlands, there is one earlier study reporting on cancer mortality among first and second generation Indonesian immigrants, which includes persons from the Moluccan Islands.[17] There seem to be multiple similarities in cancer mortality disparities between Moluccan-Dutch and the Indonesian immigrant group as a whole in the Netherlands, when compared to their Dutch counterparts. Both groups showed lower stomach cancer mortality, similar breast cancer mortality and higher liver and uterine cancer mortality when compared to their Dutch counterparts. However, the Indonesian immigrant group as a whole showed lower lung and colorectal cancer mortality when compared to native Dutch, whereas we found no differences between Moluccan-Dutch and the general Dutch population for these cancer types.

In Europe, a marked feature of cancer epidemiology in LMIC migrant groups is their lower all-cancer mortality risk.[1] However, we found that the Moluccan-Dutch experience similar all-cancer mortality when compared to the general Dutch population. Even though men showed significantly lower all-cancer mortality when compared to men in the general Dutch population, the difference was small as compared to other LMIC migrants in the Netherlands.[4,6]

Another characteristic of cancer epidemiology in LMIC migrants in Europe is a higher prevalence of cancer related to infection (pathogen-driven) and a lower prevalence of cancers thought to be related

For peer review only - http://bmjopen.bmj.com/site/about/guidelines.xhtml

to Western lifestyle (non-pathogen driven).[1] Our results imply that the Moluccan-Dutch do not fully adhere to this pattern. We will discuss all cancers for which we found a significant difference between Moluccan-Dutch and the general Dutch population and the cancer types that carried the highest mortality rates among both groups.

For cancers in which exposure to infectious agents is an important risk factor, we found higher mortality risk among Moluccan-Dutch for liver and cervical cancer and a lower mortality risk for stomach cancer, when compared to the general Dutch population.

The higher liver cancer mortality among the Moluccan-Dutch, especially in the first generation, might be reflective of early life exposure in Indonesia to higher burden of hepatitis B and C infection, the most important risk factor for liver cancer.[18] The fact that first generation migrants from Indonesia in the Netherlands show higher infectious hepatitis mortality than the general Dutch population further supports to this theory.[17] Another important risk factor for liver cancer is alcohol consumption. However, since a health survey among Moluccan-Dutch found their level of alcohol consumption to be lower when compared to native Dutch, this risk factor is unlikely to explain the difference found.[19]

Cervical cancer mortality was only significantly elevated among Moluccan-Dutch women of the second generation, when compared to their counterparts in the general Dutch population. This is in contrast with earlier research on LMIC-migrants in Europe where women of the first generation were at increased risk.[1,5] There is no data on the prevalence among Moluccan-Dutch women of the most important etiological factors for cervical cancer, specific strains of the sexually transmitted human papillomavirus.[20]

The lower stomach cancer mortality among Moluccan-Dutch was mostly attributable to a significantly lower risk in first generation adults when compared to the general Dutch population. Lower prevalence in Indonesia of the main risk factor, colonization with the bacteria Helicobacter pylori, might have had a protective effect in this group.[21,22]

For peer review only - http://bmjopen.bmj.com/site/about/guidelines.xhtml

Furthermore, we found that the Moluccan-Dutch carry higher mortality risk for cancer of the corpus uteri, but lower mortality risk for esophageal and kidney cancer and cancers of the nervous system. For cancer of the corpus uteri, the higher mortality risk among Moluccan-Dutch women when compared to women from the general Dutch population is probably not explained by an important risk factor, obesity, since obesity rate was found to be similar between the two groups.[19,23] Another important risk factor is (cumulative) estrogen exposure, but data on this topic in Moluccan-Dutch women is lacking.[24]

For esophageal cancer, important risk factors are mostly related to Western lifestyle, such as smoking, alcohol consumption and obesity.[25] The Moluccan-Dutch show no significant difference in smoking or body mass index when compared to native Dutch, but they are thought to consume less alcohol, which could contribute to the results.[19]

Kidney cancer mortality risk was lower among Moluccan-Dutch, when compared to the general Dutch population. A known risk factor for kidney cancer is hypertension, which actually may be more prevalent among Moluccan-Dutch.[15,19] Other risk factors are mostly related to a Western lifestyle, such as smoking and obesity, but prevalence of these particular risk factors is thought to be similar between Moluccan-Dutch and native Dutch.[19,26]

Cancer of the nervous system mortality risk was lower among Moluccan-Dutch than among the general Dutch population. To date, only a few factors are known to influence the incidence of gliomas, such as ionizing radiation exposure, history of atopic constitution and some genetic risk factors.[27,28] Whether these factors are able to explain the lower risk among the Moluccan-Dutch is unclear.

We found no difference in risk of mortality between the entire group of Moluccan-Dutch and the general Dutch population for the cancer types that carried the highest mortality rates.

In lung cancer, Moluccan-Dutch of the second generation showed a higher risk of dying from lung

For peer review only

cancer, when compared to the general Dutch population. Smoking is the most important risk factor of lung cancer.[29] As stated earlier, a health survey among Moluccan-Dutch found no difference in smoking between Moluccan-Dutch and native Dutch, but no stratification by generation was made.[19]

In breast cancer, Moluccan-Dutch women of the second generation experienced increased mortality risk, when compared to the general Dutch population. Since migrant women are thought to show lower attendance at the national breast cancer screening program, lower detection rate in the early stages of breast cancer, lowering the chance of curable treatment options and consequently increase mortality, might contribute to the results.[14,30] However, this raises the question why first generation Moluccan-Dutch women do not show higher mortality risk for breast cancer.

For colorectal cancer, there was no difference in the mortality risk between Moluccan-Dutch and the general Dutch population, which is in contrast with most other LMIC migrant groups in Europe.[1] One could speculate that the similar mortality risk detected in our study might be reflective of adaptation to Western lifestyle among the Moluccan-Dutch since 1951.[31]

In conclusion, several decades after migration from Indonesia to the Netherlands, the Moluccan-Dutch showed similar all-cancer mortality when compared to the general Dutch population, but differences exist in cancer-specific mortality between the two groups, in both the first and second generation. In liver and stomach cancer, an increased period of exposure to local risk factors in the Netherlands might have led to a shift in mortality towards rates of the general Dutch population. However, for most other cancer types, such a tendency was not detected, unlike some other studies in the Netherlands that included second generation migrants.[4,6] These results highlight the need for research aimed at characterizing the cancer profile of LMIC migrants in high-income countries in order to aid tailored preventative and diagnostic efforts.

For peer review only - http://bmjopen.bmj.com/site/about/guidelines.xhtml

References

1. Arnold, M., Razum, O. & Coebergh, J. W. Cancer risk diversity in non-western migrants to Europe: An overview of the literature. *Eur. J. Cancer* **46**, 2647–2659 (2010).
2. Gushulak, B. D. & MacPherson, D. W. The basic principles of migration health: Population mobility and gaps in disease prevalence. *Emerg. Themes Epidemiol.* **3**, 1–11 (2006).
3. Spallek, J., Zeeb, H. & Razum, O. What do we have to know from migrant ' s past exposures to understand their health status ? A life course approach. *Emerg. Themes Epidemiol.* **8**, 1–8 (2011).
4. Stirbu, I. *et al.* Cancer mortality rates among first and second generation migrants in the Netherlands: Convergence toward the rates of the native Dutch population. *Int. J. Cancer* **119**, 2665–2672 (2006).
5. Beiki, O., Allebeck, P., Nordqvist, T. & Moradi, T. Cervical, endometrial and ovarian cancers among immigrants in Sweden: Importance of age at migration and duration of residence. *Eur. J. Cancer* **45**, 107–118 (2009).
6. Williams, G. *et al.* Cancer incidence and mortality of Surinamese migrants in the Netherlands: In-between Surinamese and Dutch levels? *Cancer Causes Control* **24**, 1375–1383 (2013).
7. van Amersfoort, H. The waxing and waning of a diaspora: Moluccans in the Netherlands, 1950-2002. *J. Ethn. Migr. Stud.* **30**, 151–174 (2004).
8. Arnold, M., Aarts, M. J., Van Der Aa, M., Visser, O. & Coebergh, J. W. Investigating cervical, oesophageal and colon cancer risk and survival among migrants in the Netherlands. *Eur. J. Public Health* **23**, 867–873 (2013).
9. Arnold, M. *et al.* Diverging breast and stomach cancer incidence and survival in migrants in the Netherlands, 1996-2009. *Acta Oncol. (Madr).* **52**, 1195–1201 (2013).
10. Visser, O. & van Leeuwen, F. E. Cancer risk in first generation migrants in North-Holland/Flevoland, The Netherlands, 1995-2004. *Eur. J. Cancer* **43**, 901–908 (2007).

For peer review only - http://bmjopen.bmj.com/site/about/guidelines.xhtml

For peer review only

11. Torre, L. A. *et al.* Global Cancer Statistics, 2012. *CA a cancer J. Clin.* **65**, 87–108 (2015).

12. Bodewes, A. J., Agyemang, C. & Kunst, A. E. All-cause mortality among three generations of Moluccans in the Netherlands. *Eur. J. Public Health* **29**, 463–467 (2018).

13. Bodewes, A. J. & Kunst, A. E. Involving hard-to-reach ethnic minorities in low-budget health research: Lessons from a health survey among Moluccans in the Netherlands. *BMC Res. Notes* **9**, 1–8 (2016).

14. Vermeer, B. & Van den Muijsenbergh, M. E. T. C. The attendance of migrant women at the national breast cancer screening in the Netherlands 1997-2008. *Eur. J. Cancer Prev.* **19**, 195–8 (2010).

15. De Back, T. R., Bodewes, A. J., Brewster, L. M. & Kunst, A. E. Cardiovascular health and related health care use of Moluccan-Dutch immigrants. *PLoS One* **10**, 1–11 (2015).

16. Verhagen, I., Ros, W. J. G., Steunenberg, B., Laan, W. & De Wit, N. J. Differences in health care utilisation between elderly from ethnic minorities and ethnic Dutch elderly. *Int. J. Equity Health* **13**, 1–7 (2014).

17. Ho, L., Bos, V. & Kunst, A. E. Differences in cause-of-death patterns between the native Dutch and persons of Indonesian descent in the Netherlands. *Am. J. Public Health* **97**, 1616–1618 (2007).

18. El-Serag, H. B. Epidemiology of viral hepatitis and hepatocellular carcinoma. *Gastroenterology* **142**, 1264–1273.e1 (2012).

19. Van Der Wal, J. M., Bodewes, A. J., Agyemang, C. O. & Kunst, A. E. Higher self-reported prevalence of hypertension among Moluccan-Dutch than among the general population of the Netherlands: Results from a cross-sectional survey. *BMC Public Health* **14**, (2014).

20. Schiffman, M., Castle, P., Jeronimo, J., Rodriguez, A. & Wacholder, S. Human papillomavirus and cervical cancer. *Lancet* **370**, 890–907 (2007).

21. Huang, H. *et al.* Estimation of Cancer Burden Attributable to Infection in Asia. *J. Epidemiol.* **25**, 626–638 (2015).

For peer review only

For peer review only - http://bmjopen.bmj.com/site/about/guidelines.xhtml

22. Blankenstein, M. Van *et al.* The prevalence of helicobacter pylori infection in the Netherlands. **48**, (2013).

23. Reeves, G. K. *et al.* Cancer incidence and mortality in relation to body mass index in the Million Women Study: Cohort study. *Br. Med. J.* **335**, 1134–1139 (2007).

24. Collaborative Group on Epidemiological Studies on Endmetrial Cancer. Endometrial cancer and oral contraceptives: An individual participant meta-analysis of 27 276 women with endometrial cancer from 36 epidemiological studies. *Lancet Oncol.* **16**, 1061–1070 (2015).

25. Thrift, A. P. The epidemic of oesophageal carcinoma: Where are we now? *Cancer Epidemiol.* **41**, 88–95 (2016).

26. Weikert, S. & Ljungberg, B. Contemporary epidemiology of renal cell carcinoma: Perspectives of primary prevention. *World J. Urol.* **28**, 247–252 (2010).

27. Ostrom, Q. T. *et al.* The epidemiology of glioma in adults: A state of the science review. *Neuro. Oncol.* **16**, 896–913 (2014).

28. Maile, E. J., Barnes, I., Finlayson, A. E., Sayeed, S. & Ali, R. Nervous system and intracranial tumour incidence by ethnicity in England, 2001-2007: A descriptive epidemiological study. *PLoS One* **11**, 2001–2007 (2016).

29. Alberg, A. J., Brock, M. V., Ford, J. G., Samet, J. M. & Spivack, S. D. Epidemiology of lung cancer: Diagnosis and management of lung cancer, 3rd ed: American college of chest physicians evidence-based clinical practice guidelines. *Chest* **143**, 1–29 (2013).

30. Islanders, P. *et al.* Differences in Breast Cancer Stage, Treatment, and Survival by Race and Ethnicity. **163**, 1–7 (2003).

31. Rasool, S., Kadla, S. A., Rasool, V. & Ganai, B. A. A comparative overview of general risk factors associated with the incidence of colorectal cancer. *Tumor Biol.* **34**, 2469–2476 (2013).

For peer review only

List of abbreviations

LMIC: low- and middle income countries

HIC: high-income countries

KNIL: Royal Netherlands East Indies Army

CBS: Statistics Netherlands

SES: social economic status

SCP: Netherlands Institute for Social Research

ICD-10: 10th revision of the International Statistical Classification of Diseases and Related Health Problems

CI: confidence interval

RR: relative risk

NHL: non-Hodgkin lymphoma

For peer review only

For peer review only - http://bmjopen.bmj.com/site/about/guidelines.xhtml

Table 1: Demographic characteristics the Moluccan-Dutch and the Dutch general population				
	Moluccan-Dutch		Dutch general population	
	N	%	N	%
Sex				
Male	40062	49.1	7917402	49.9
Female	41529	50.9	7949136	50.1
Generation (birth year)				
1 adult (<1934)	4642	5.7	2480434	15.6
1 minor (1935-1951)	8185	10.0	2639408	16.6
2 (1951-1969)	22754	27.9	3944327	24.9
≥2 (1970 >)	46010	56.4	6802369	42.9
Decile score				
1 (lowest SES[a])	7017	8.6	729861	4.6
2	7669	9.4	951992	6.0
3	10363	12.7	1729453	10.9
4	11341	13.9	1745319	11.0
5	11096	13.6	2491047	15.7
6	6935	8.5	1919851	12.1
7	6283	7.7	1856385	11.7
8	4732	5.8	1174124	7.4
9	7262	8.9	1586654	10.0
10 (highest SES)	8648	10.6	1650120	10.4
Missing	245	0.3	31732	0.2
Total	81591	100	15866538	100

a: SES = socioeconomic status

For peer review only - http://bmjopen.bmj.com/site/about/guidelines.xhtml

Table 2: Absolute numbers of death and age-standardized death rates per cancer type					
		Absolute numbers of death		Age-standardized death rates per 100,000	
Type of cancer	ICD-10[a] code	Dutch general population	Moluccan-Dutch	Dutch general population	Moluccan-Dutch
Stomach	C16	18443	33	4.98	3.51
Liver cell	C22	7227	53	2.03	6.37
Cervix uteri	C53	2488	15	0.77	1.51
Colorectal	C18-21	59548	138	16.11	15.51
Breast	C50	41324	134	12.59	13.20
Prostate	C61	30975	79	7.60	10.93
Bladder	C67	14875	27	3.86	2.91
Lung and lower airway	C33-34	119520	336	34.67	34.89
Esophagus	C15	18683	25	5.51	2.55
Corpus uteri	C54	5189	22	1.38	2.15
Ovaries	C56	12239	40	3.52	3.76
Pancreas	C25	27351	62	7.64	6.95
Kidney	C64	11491	20	3.24	1.98
Leukemia	C91-95	14349	48	3.71	5.04
Non-Hodgkin lymphoma	C82-85, C96	13620	48	3.70	5.13
Nervous-system	C70-72	10893	19	3.36	1.07

a: ICD-10 = 10th revision of the International Statistical Classification of Diseases and Related Health Problems

For peer review only

Table 3: Poisson regression for cancer-specific mortality and all-cancer mortality among Moluccan-Dutch, adjusted for age-at-baseline and socioeconomic status.

Cancer type	Both sexes		Males		Females	
	N	RR[a] (95% CI[b])	N	RR (95% CI)	N	RR (95% CI)
Stomach	33	0.71 (0.50 – 1.00)	17	0.57 (0.35 – 0.91)	16	0.96 (0.59 – 1.57)
Liver	53	2.60 (1.98 – 3.42)	33	2.58 (1.83 – 3.64)	20	2.65 (1.69 – 4.17)
Cervix	15	-	-	-	15	1.89 (1.13 – 3.14)
Colorectal	138	0.95 (0.80 – 1.12)	81	1.01 (0.81 – 1.26)	57	0.87 (0.67 – 1.13)
Breast	134	-	-	-	134	1.08 (0.91 – 1.29)
Prostate	79	-	79	1.21 (0.97 – 1.51)	-	-
Bladder	27	0.75 (0.51 – 1.10)	16	0.64 (0.39 – 1.05)	11	1.03 (0.55 – 1.91)
Lung and lower airway	336	0.95 (0.85 – 1.05)	201	0.90 (0.78 – 1.03)	135	1.00 (0.84 – 1.19)
Esophagus	25	0.46 (0.31 – 0.68)	23	0.55 (0.37 – 0.83)	<10	0.15 (0.04 – 0.62)
Corpus uteri	22	-	-	-	22	1.72 (1.12 – 2.64)
Ovaries	40	-	-	-	40	1.19 (0.87 – 1.63)
Pancreas	62	0.86 (0.67 – 1.11)	24	0.63 (0.42 – 0.95)	38	0.13 (0.82 – 1.55)
Kidney	20	0.61 (0.39 – 0.96)	14	0.70 (0.41 – 1.18)	<10	0.46 (0.19 – 1.11)
Leukemia	48	1.21 (0.89 – 1.65)	25	1.17 (0.77 – 1.76)	23	1.27 (0.80 – 2.02)
Non-Hodgkin lymphoma	48	1.29 (0.96 – 1.73)	35	1.60 (1.13 – 2.27)	13	0.84 (0.48 – 1.49)
Nervous system	19	0.32 (0.18 – 0.58)	11	0.30 (0.13 – 0.66)	<10	0.36 (0.15 – 0.86)
All cancers (ICD-10[d] codes C00 – D48)		0.98 (0.93 – 1.03)		0.92 (0.85 – 0.99)		1.05 (0.97 – 1.13)

a: RR = relative risk; b: CI = confidence interval; c: ICD-10 = 10th revision of the International Statistical Classification of Diseases and Related Health Problems. N < 10 is not shown in accordance with Statistics Netherlands (CBS) guidelines.

For peer review only

Table 4: Poisson regression for cancer mortality among Moluccan-Dutch stratified by generation, adjusted for age-at-baseline, sex and socioeconomic status

Cancer type	Generation					
		1 (adult)		1 (minor)		2
	N	RR[a] (95% CI[b])	N	RR (95% CI)	N	RR (95% CI)
Stomach	10	0.48 (0.26 – 0.89)	10	0.65 (0.35 – 1.21)	13	1.27 (0.73 – 2.19)
Liver	29	3.96 (2.74 – 5.70)	14	1.79 (1.06 – 3.03)	<10	1.86 (0.96 – 3.59)
Cervix	<10	1.72 (0.55 – 5.34)	<10	0.91 (0.23 – 3.64)	10	2.39 (1.28 – 4.47)
Colorectal	67	1.06 (0.83 – 1.34)	48	0.92 (0.69 – 1.22)	23	0.75 (0.50 – 1.13)
Breast	24	0.79 (0.53 – 1.17)	42	1.05 (0.77 – 1.42)	65	1.29 (1.01 – 1.65)
Prostate	71	1.61 (1.28 – 2.03)	<10	0.45 (0.22 – 0.90)	-	-
Bladder	11	0.58 (0.32 – 1.05)	<10	0.48 (0.20 – 1.16)	10	1.81 (0.97 – 3.38)
Lung and lower airway	99	0.82 (0.68 – 1.01)	99	0.70 (0.58 – 0.85)	135	1.42 (1.20 – 1.69)
Esophagus	<10	0.37 (0.17 – 0.82)	<10	0.39 (0.21 – 0.76)	10	0.63 (0.34 – 1.16)
Corpus uteri	<10	1.21 (0.54 – 2.69)	<10	1.52 (0.71 – 3.17)	<10	3.14 (1.56 – 6.33)
Ovaries	<10	0.67 (0.32 – 1.40)	15	1.18 (0.71 – 1.96)	17	1.76 (1.09 – 2.83)
Pancreas	29	1.09 (0.76 – 1.57)	23	0.81 (0.54 – 1.21)	10	0.59 (0.31 – 1.09)
Kidney	<10	0.52 (0.23 – 1.15)	<10	0.43 (0.18 – 1.04)	<10	1.01 (0.50 – 2.02)
Leukemia	17	1.14 (0.71 – 1.84)	<10	0.79 (0.41 – 1.51)	15	1.95 (1.17 – 3.25)
Non-Hodgkin lymphoma	24	1.68 (1.13 – 2.51)	12	0.98 (0.55 – 1.72)	<10	1.03 (0.51 – 2.07)
Nervous system	<10	0.36 (0.09 – 1.44)	<10	0.39 (0.16 – 0.93)	<10	0.25 (0.09 – 0.66)

a: RR = relative risk; b: CI = confidence interval. N < 10 is not shown in accordance with Statistics Netherlands (CBS) guidelines.

For peer review only - http://bmjopen.bmj.com/site/about/guidelines.xhtml

Reporting checklist for cohort study.

Based on the STROBE cohort guidelines.

Instructions to authors

Complete this checklist by entering the page numbers from your manuscript where readers will find each of the items listed below.

Your article may not currently address all the items on the checklist. Please modify your text to include the missing information. If you are certain that an item does not apply, please write "n/a" and provide a short explanation.

Upload your completed checklist as an extra file when you submit to a journal.

In your methods section, say that you used the STROBE cohort reporting guidelines, and cite them as:

von Elm E, Altman DG, Egger M, Pocock SJ, Gotzsche PC, Vandenbroucke JP. The Strengthening the Reporting of Observational Studies in Epidemiology (STROBE) Statement: guidelines for reporting observational studies.

		Reporting Item	Page Number
Title	#1a	Indicate the study's design with a commonly used term in the title or the abstract	1
Abstract	#1b	Provide in the abstract an informative and balanced summary of what was done and what was found	3

For peer review only

Background / rationale	#2	Explain the scientific background and rationale for the investigation being reported	5-6
Objectives	#3	State specific objectives, including any prespecified hypotheses	6
Study design	#4	Present key elements of study design early in the paper	7-8
Setting	#5	Describe the setting, locations, and relevant dates, including periods of recruitment, exposure, follow-up, and data collection	7-8
Eligibility criteria	#6a	Give the eligibility criteria, and the sources and methods of selection of participants. Describe methods of follow-up.	7-8
	#6b	For matched studies, give matching criteria and number of exposed and unexposed	n/a
Variables	#7	Clearly define all outcomes, exposures, predictors, potential confounders, and effect modifiers. Give diagnostic criteria, if applicable	7-8
Data sources / measurement	#8	For each variable of interest give sources of data and details of methods of assessment (measurement). Describe comparability of assessment methods if there is more than one group. Give information separately for for exposed and unexposed groups if applicable.	7-8
Bias	#9	Describe any efforts to address potential sources of bias	12
Study size	#10	Explain how the study size was arrived at	7-10
Quantitative	#11	Explain how quantitative variables were handled in the	7-8

For peer review only

variables		analyses. If applicable, describe which groupings were chosen, and why	
Statistical methods	#12a	Describe all statistical methods, including those used to control for confounding	7-8
	#12b	Describe any methods used to examine subgroups and interactions	7-8
	#12c	Explain how missing data were addressed	n/a
	#12d	If applicable, explain how loss to follow-up was addressed	n/a
	#12e	Describe any sensitivity analyses	n/a
Participants	#13a	Report numbers of individuals at each stage of study—eg numbers potentially eligible, examined for eligibility, confirmed eligible, included in the study, completing follow-up, and analysed. Give information separately for for exposed and unexposed groups if applicable.	7-8, 10
	#13b	Give reasons for non-participation at each stage	n/a
	#13c	Consider use of a flow diagram	n/a
Descriptive data	#14a	Give characteristics of study participants (eg demographic, clinical, social) and information on exposures and potential confounders. Give information separately for exposed and unexposed groups if applicable.	10
	#14b	Indicate number of participants with missing data for each variable of interest	21

For peer review only

	#14c	Summarise follow-up time (eg, average and total amount)	n/a
Outcome data	#15	Report numbers of outcome events or summary measures over time. Give information separately for exposed and unexposed groups if applicable.	10-11
Main results	#16a	Give unadjusted estimates and, if applicable, confounder-adjusted estimates and their precision (eg, 95% confidence interval). Make clear which confounders were adjusted for and why they were included	10-11
	#16b	Report category boundaries when continuous variables were categorized	7-8
	#16c	If relevant, consider translating estimates of relative risk into absolute risk for a meaningful time period	n/a
Other analyses	#17	Report other analyses done—e.g., analyses of subgroups and interactions, and sensitivity analyses	10-11
Key results	#18	Summarise key results with reference to study objectives	12
Limitations	#19	Discuss limitations of the study, taking into account sources of potential bias or imprecision. Discuss both direction and magnitude of any potential bias.	12
Interpretation	#20	Give a cautious overall interpretation considering objectives, limitations, multiplicity of analyses, results from similar studies, and other relevant evidence.	12-16
Generalisability	#21	Discuss the generalisability (external validity) of the study	12, 16

For peer review only

		results	
Funding	#22	Give the source of funding and the role of the funders for the present study and, if applicable, for the original study on which the present article is based	2

The STROBE checklist is distributed under the terms of the Creative Commons Attribution License CC-BY. This checklist can be completed online using https://www.goodreports.org/, a tool made by the EQUATOR Network in collaboration with Penelope.ai

For peer review only
